# Generating Question Prompt Lists From Electronic Health Record Data Using Large Language Models: Iterative Evaluation Study

**DOI:** 10.2196/87280

**Published:** 2026-07-09

**Authors:** Zhe He, Balu Bhasuran, Mia Liza A Lustria, Karim Hanna, Michael Killian, Cindy Shavor, Mandy Dailey, Sai Sidharth Manikandan, Xiao Luo

**Affiliations:** 1 School of Information, Florida State University, 142 Collegiate Loop, Tallahassee, FL, 32306, United States, 1 8506445775; 2 Department of Family Medicine, Morsani College of Medicine, University of South Florida, Tampa, FL, United States; 3 College of Social Work, Florida State University, Tallahassee, FL, United States; 4 Department of Statistics, Florida State University, Tallahassee, FL, United States; 5 Department of Computer Science, Southern Methodist University, Dallas, TX, United States

**Keywords:** large language models, laboratory results interpretation, electronic health records, question prompt lists, patient-clinician communication

## Abstract

**Background:**

Patients frequently access laboratory results through patient portals, but many struggle to interpret these values and formulate relevant questions for their clinicians. Question prompt lists (QPLs) can enhance communication but are rarely tailored to individual clinical contexts.

**Objective:**

This study evaluated the feasibility of using large language models (LLMs) to generate patient-friendly, clinically relevant questions grounded in electronic health record (EHR) laboratory data.

**Methods:**

We extracted deidentified clinical profiles, including laboratory results, diagnoses, and medications, from patients with chronic conditions (eg, diabetes and chronic kidney disease). Using 9 deidentified clinical profiles from the OneFlorida Data Trust, we generated 486 questions across all rounds: 126 with GPT-4o in round 1, 120 with GPT-4o in round 2, 180 with GPT-4o in round 3, and 60 with LLaMA 3.2 in round 3. Prompt refinements were informed by clinician ratings consisting of 2 binary questions (ie, clear phrasing and clinical validity) and 3 Likert-scale questions (ie, significance for the patient’s health; clinical appropriateness, that is, the likelihood of being asked in primary care setting; and willingness to answer). Refinements were incorporated after each round. Patient participants then evaluated selected questions for understandability, perceived usefulness, and intention to use. Readability was assessed with standard indices.

**Results:**

Iterative clinician feedback improved question clarity and reduced clinically irrelevant suggestions. Across rounds, GPT-4o consistently produced more coherent and patient-friendly questions, while LLaMA 3.2 demonstrated competitive performance on Likert-scale metrics. It exhibited greater variability in clinical appropriateness as noted by clinicians. In round 3, the binary metric “clear phrasing” reached a ceiling effect for both models, while clinical validity ratings showed greater variability, particularly from one clinician. Likert-scale evaluations tended to favor LLaMA 3.2 across all 3 clinicians for clinical appropriateness (3.37‐4.82 vs 3.02‐4.67), significance for the patient’s health (3.38‐4.28 vs 2.97‐3.83), and willingness to answer (3.17‐4.70 vs 2.82‐4.47), with multiple comparisons reaching statistical significance after Bonferroni correction. Patient evaluation (N=134) of GPT-4o–generated questions showed that 25 of 30 questions had moderate to high understandability (average Likert score ≥3.5), and 19 of 30 questions had moderate to high usefulness (average Likert score ≥3.5).

**Conclusions:**

This study supports the feasibility of using LLMs with structured EHR-derived laboratory data to generate contextualized QPLs, but model outputs varied in clinical appropriateness and readability. Clinician-in-the-loop review remains necessary before patient-facing use.

## Introduction

The 21st Century Cures Act and subsequent regulations on information blocking require health systems to provide patients with near real-time access to their test results, clinical notes, and other records through certified electronic health record (EHR) systems [1]. These policies build on earlier Health Information Technology for Economic and Clinical Health (HITECH) Act incentives that drove widespread EHR adoption and patient portal implementation. Patient portals have made laboratory results readily available to patients, and viewing laboratory results is one of the most used features of patient portals [2,3]. Millions of patients now receive laboratory results directly through portals, often before a clinician can contextualize them. Yet, access alone rarely translates into understanding: laboratory values are typically presented in clinician-oriented tables, often without explanations that distinguish “near-normal” from clinically significant results, which can lead to misinterpretation and missed opportunities for timely follow-up [4]. These challenges disproportionately affect older adults, people with multiple chronic conditions, and those with limited health literacy.

Question prompt lists (QPLs)—structured questions that patients bring to a visit—consistently improve recall, communication, and shared decision-making [5,6]. However, most QPLs are static, paper based, and generic—limiting their ability to reflect a patient’s current clinical context (eg, abnormal laboratory results, active diagnoses, and medications). A digital approach that tailors QPLs to each patient’s medical profile and laboratory results could better focus limited visit time on what matters most.

Recent advances in AI-based large language models (LLMs) have opened new avenues for enhancing patient education, diagnostics, and medical Q&A, among others [7-12]. LLMs are advanced AI systems that use deep learning techniques to process and generate natural language (eg, ChatGPT developed by OpenAI). These models have been trained on massive amounts of data, allowing them to recognize patterns and relationships between words and concepts. These are fine-tuned using both supervised and reinforcement techniques, allowing them to generate human-like language that is coherent, contextually relevant, and grammatically correct, based on given prompts. While LLMs such as ChatGPT have gained popularity, a recent study by the European Federation of Clinical Chemistry and Laboratory Medicine (EFLM) working group on AI showed that these may give superficial or even incorrect answers to laboratory test results–related questions and thus cannot be used for diagnosis [13]. In our previous work, we found that LLMs such as GPT-4 could generate significantly more accurate, helpful, safe, and relevant answers to laboratory result questions posted by patients than the answers made by peer patients on general social Q&A websites such as Yahoo! Answers [7]. It is yet unknown if LLMs can automatically generate patient-friendly and clinically meaningful questions based on patients’ EHR data.

LLMs have been increasingly investigated for their potential to improve patient-clinician communication, particularly through the support of clinician messaging workflows [9]. Several studies have shown that GPT-4 and related models can generate portal message drafts rated higher than physician-authored replies in empathy, tone, and communication quality, while remaining comparable in accuracy. In a blinded study of real patient exchanges, physicians judged LLM-generated responses as clearer and more compassionate, and iterative prompt engineering further improved clinician acceptance of AI-drafted replies [14]. Large-scale deployments such as PAM Chat**,** which generated over 20,000 nurse-reviewed portal replies, demonstrated that AI-assisted messaging can accelerate response times, reduce clinician burden, and even enhance patient satisfaction scores [15]. Similarly, institution-specific fine-tuned models such as Comprehensive Large Language Model Artificial Intelligence Responder (CLAIR)-Long model (an LLaMA-2–based system trained on nearly half a million patient-provider messages) achieved performance on par with commercial LLMs while better reflecting the tone and style of local clinical communication [9]. Collectively, this body of work suggests that LLMs can play a meaningful role in reducing clinician workload while sustaining or improving patient experience.

More recently, researchers have shifted attention upstream, asking whether LLMs can also support patients in composing clearer, more complete communications. For example, Liu et al [16] demonstrated that generating suggested follow-up questions before message submission reduced ambiguity and the need for back-and-forth exchanges while maintaining provider relevance. FollowupQ, a multiagent framework, synthesized EHR data and patient portal messages to propose personalized follow-up questions, significantly reducing provider workload and aligning more closely with ground-truth clinical inquiries [17]. Other approaches have leveraged in-context learning and few-shot prompting to train LLMs to elicit targeted, diagnostic reasoning–driven questions during medical preconsultation, enabling more comprehensive patient histories before a clinical encounter [18]. These early explorations illustrate how LLMs could enhance not only the clinician side of messaging but also the quality and completeness of patient-initiated communication.

Despite these advances, little work has focused on tailoring question generation to structured clinical data, such as laboratory test results. Patients most often access portals to view laboratory results, yet prior systems have rarely attempted to transform those results into actionable, patient-centered question prompts. Current LLM-based interventions also risk producing generic or clinically irrelevant suggestions when decoupled from context.

This study addresses that gap by evaluating an LLM-based framework that generates patient-friendly, clinically meaningful question prompts grounded in EHR-derived laboratory data and aligned with active conditions and medications. Unlike earlier approaches focused on message reply drafting or general inquiry generation, our work emphasizes clinician-reviewed, lab-anchored, patient-facing prompts, designed to enhance understanding and guide productive conversations in time-limited primary care visits. We used a user-centered, iterative process with board-certified family physicians to refine prompts for clarity, clinical validity, clinical appropriateness, and prioritization by urgency; then assessed patient perceptions of understandability and usefulness; and assessed the readability of the prompts. This study advances the design and evaluation of LLM-based communication tools in 3 ways. First, it demonstrates a practical method for transforming structured laboratory data into tailored QPLs enabling patients to receive context-specific questions that support more informed clinical conversations. Second, it establishes an iterative, clinician-in-the-loop prompt engineering protocol that reduces hallucinations and strengthens the clinical actionability of model outputs to provide a pathway toward safer integration of AI in health care. Lastly, through a mixed methods evaluation of 2 LLMs (ie, GPT-4o and LLaMA 3.2), it provides useful evidence on the clarity, relevance, readability, and patient-perceived understandability and usefulness of LLM-generated content, to help inform the design of more effective models for QPL generation.

## Methods

### Study Design

**Figure 1.**
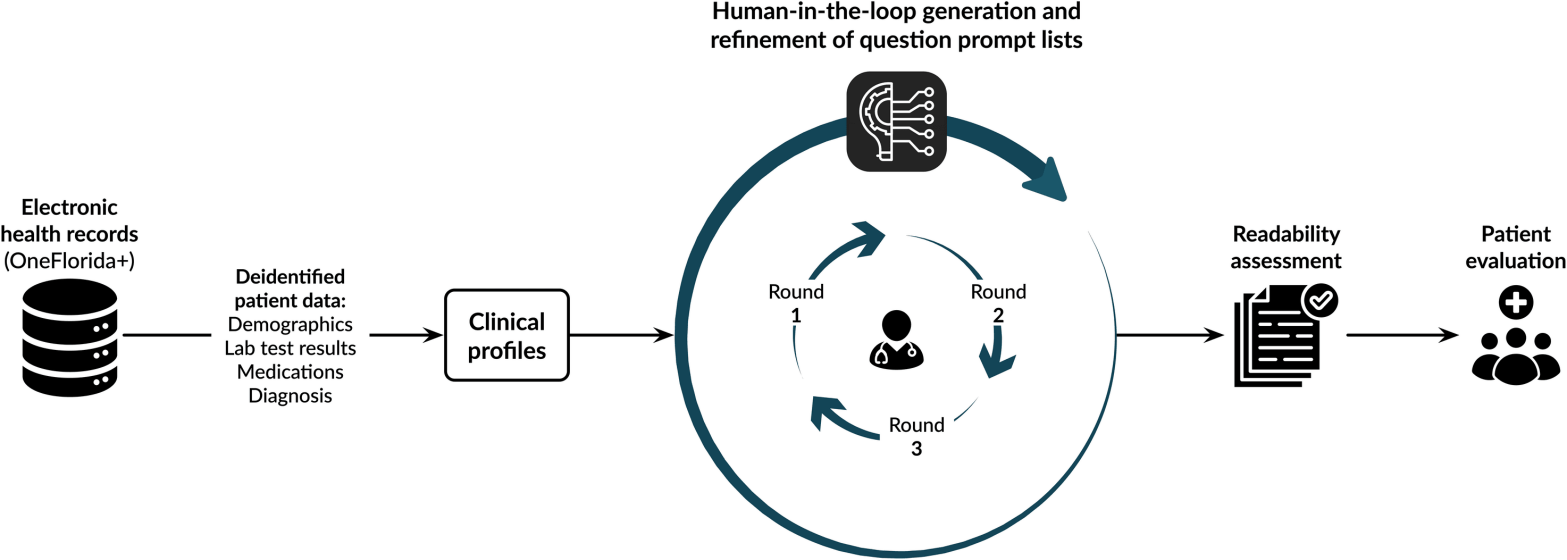
Overall study flow. The study used deidentified patient data from electronic health records (OneFlorida+) to construct clinical profiles containing demographics, laboratory test results, medications, and diagnoses. Large language models (LLMs) were applied to generate tailored question prompt lists (QPLs) through a 3-round, human-in-the-loop refinement process with clinician input. After iterative evaluation and revision, the generated questions underwent readability assessment using standard indices and were subsequently rated by patients for understandability and perceived usefulness. This workflow illustrates the integration of EHR data, clinician feedback, and patient evaluation to assess the feasibility of LLM-assisted QPL generation.

We evaluated the feasibility of using LLMs to generate patient-centered, clinically relevant questions grounded in laboratory data through a multistage process. First, we constructed deidentified clinical profiles using EHR data that included laboratory results, diagnoses, medications, and demographics. Second, we refined the generation process through 3 rounds of clinician-in-the-loop review, during which physicians evaluated LLM outputs for clinical clarity, clinical validity, clinical appropriateness, significance for the patient’s health, and willingness to answer. Readability metrics were then applied to the refined outputs. Finally, a subset of questions was organized into 3 hypothetical patient profiles, and a web-based survey with adult patients (N=134) was conducted to assess the clarity and usefulness of these AI-generated prompts. The overall workflow is shown in Figure 1, with full methodological details provided in the *Methods* section. The following subsections present the results from each phase of the study.

### Ethical Considerations

The study was approved by the Florida State University Institutional Review Board with an umbrella study record number STUDY00004785. The retrospective patient data were derived from deidentified OneFlorida+ EHR data. The use of OneFlorida+ EHR data was approved with a study record number STUDY00000483. As the clinical data for these patients were deidentified and used retrospectively, informed consent was waived by the institutional review board for this component of the study. The study for question evaluation by providers and patients was approved with a study record number STUDY00005926. Written informed consent was obtained from all participants in the prospective survey evaluation before participation. Survey participants were informed about the study purpose, procedures, potential risks, and benefits before they provided consent.

### Case Selection

Clinical profiles were derived from deidentified OneFlorida+ EHR data with all dates shifted or removed to protect privacy. We first filtered records to include patients with Alzheimer disease (International Classification of Diseases, 9th revision [ICD-9], code 331.0; International Classification of Diseases, 10th revision [ICD-10], code G30.x), type 2 diabetes mellitus (ICD-9 codes 250.x0, 250.x2; ICD-10 code E11.x), or chronic kidney disease (ICD-9 code 585.x; ICD-10 code N18.x). Laboratory data were restricted to outpatient encounters (eg, primary care and geriatric clinics) to reflect routine care rather than acute admissions. Profiles included demographics, laboratory tests, diagnoses, and medications. Laboratory results were mapped to Logical Observation Identifiers Names and Codes (LOINC), medications standardized with RxNorm Concept Unique Identifiers (CUIs), and diagnoses coded in ICD-9 or ICD-10. To ensure recency, only the most recent laboratory results were retained, along with medications and diagnoses documented within 6 months of the laboratory test date. Patients with abnormal values for hemoglobin A_1c_ (HbA_1c_), creatinine, or estimated glomerular filtration rate (eGFR) were prioritized. Gender-specific reference ranges were applied for creatinine (0.7‐1.3 mg/dL for men and 0.5‐1.1 mg/dL for women), and standard thresholds were used for HbA_1c_ (4%‐5.6%), eGFR (60‐120 mL/min/1.73 m^2^), urea nitrogen to creatinine ratio (10-20), and creatine kinase (30‐200 U/L). This standardized approach ensured that each profile reflected clinically relevant, up-to-date patient information suitable for question generation.

### LLM Selection and Iterative Evaluation by Clinicians

Two LLMs were evaluated: GPT-4o (OpenAI API) [19] and LLaMA 3.2 [20] (3B Instruct Q8, accessed locally via Jan [21]). We evaluated GPT-4o and a quantized LLaMA 3.2 3B model to reflect both proprietary and deployment-feasible open-source settings. The LLaMA 3.2 3B model was selected based on anticipated real-world deployment constraints, including low latency, computational efficiency, and the ability to run locally or on edge infrastructure to support privacy-preserving workflows. Larger open-source models, while offering greater reasoning capacity, would introduce substantially higher latency and hardware requirements that are misaligned with real-time, patient-facing applications. Accordingly, this study does not aim to benchmark open-source LLMs at an equivalent scale but to assess whether a deployment-feasible model can generate clinically acceptable question prompts. Both models were configured with deterministic parameters (temperature=0, top-p=1) to ensure reproducibility and comparability.

The evaluation was conducted in 3 rounds. In round 1**,** GPT-4o generated questions for 3 diverse profiles (ie, a Hispanic woman aged ≥90 years, a Hispanic woman aged 83 years, and a White man aged 78 years). Board-certified family physicians reviewed outputs for clarity, clinical validity, clinical appropriateness (ie, the likelihood of being asked by patients in primary care setting), significance for the patient’s health, and willingness to answer. In rounds 2 and 3, prompts were iteratively refined based on feedback from clinicians. Six profiles were used, each paired with a counterpart with different demographics to test robustness. In round 3, the previously used 6 profiles and 3 entirely new profiles (eg, a White woman aged 74 years, an African American woman aged 79 years, and a White man aged 69 years) were used to generate questions with GPT-4o. To compare the performance of GPT-4o and LLaMA 3.2, we also used LLaMA 3.2 to generate questions using the 3 new profiles. This design allowed a comparative evaluation of the 2 models in terms of clarity, contextual accuracy, and adaptability to novel patient scenarios. Table 1 lists the clinical profiles used in each round of prompt engineering with clinician evaluation. Table S1 in Multimedia Appendix 1 contains an example synthetic patient profile used for question generation. The actual patient-level data cannot be shared publicly due to the Health Insurance Portability and Accountability Act (HIPAA) regulation.

**Table 1.** Clinical profiles used in each round of prompt engineering with clinician evaluation^a^.

Patient profile	Clinical profile description	Round 1 GPT-4o	Round 2 GPT-4o	Round 3 GPT-4o	Round 3 LLaMA 3.2
Patient #1	Hispanic woman aged ≥90 years	✓	✓	✓	—^b^
Patient #2	Hispanic woman aged 83 years	✓	✓	✓	—
Patient #3	White man aged 78 years	✓	✓	✓	—
Patient #4	White woman aged 75 years	—	✓	✓	—
Patient #5	African American man aged 73 years	—	✓	✓	—
Patient #6	African American woman aged 82 years	—	✓	✓	—
Patient #7	White woman aged 74 years	—	—	✓	✓
Patient #8	African American woman aged 79 years	—	—	✓	✓
Patient #9	White man aged 69 years	—	—	✓	✓

a ✓ indicates usage in the corresponding round.

b Not applicable.

### Prompt Engineering

To generate clinically meaningful and patient-friendly questions about laboratory test results, we used an iterative prompt engineering process using LLMs. This process unfolded over 3 sequential rounds, each guided by feedback from board-certified family physicians. In each round, questions were generated from structured, EHR-derived clinical profiles using an initial prompt template. Outputs were then evaluated for clarity, clinical relevance, and primary care applicability. Based on clinician feedback, the prompts were refined to improve specificity, readability, and alignment with patients’ active medical conditions and medications. This iterative approach progressively optimized question quality, reduced hallucinations, and enhanced the overall usability of the generated questions for real-world patient-provider communication.

### Evaluation Framework

#### Evaluation by Clinicians

We evaluated the quality and clinical utility of the LLM-generated questions through a 3-round iterative process involving 3 board-certified family physicians. In each round, physicians independently rated the generated questions across five dimensions: (1) clarity (ie, clear phrasing; yes/no), (2) clinical validity (yes/no), (3) clinical appropriateness (ie, the likelihood of being asked in primary care setting; 1‐5 Likert scale), (4) significance for the patient’s health (1‐5 Likert scale), and (5) willingness to answer (1‐5 Likert scale). After round 1, semistructured interviews were conducted to obtain in-depth qualitative feedback. These interviews were transcribed and analyzed thematically using NVivo (version 15; Lumivero) to identify the common strengths and areas for improvement, which informed the subsequent rounds of prompt refinement. In rounds 2 and 3, all physicians evaluated the same set of generated questions, enabling the calculation of interrater reliability using intraclass correlation coefficients (ICCs). This mixed methods evaluation approach ensured both quantitative rigor and qualitative insight into the clarity, clinical validity, and clinical appropriateness of the questions for primary care contexts.

#### Readability Assessment

To assess the objective readability of LLM-generated clinical questions, we also applied a suite of standard readability metrics using the TextStat Python library. These measures provide quantitative estimates of linguistic complexity and reading difficulty, allowing a comparison of outputs from GPT-4o and LLaMA 3.2. For each question, we computed the Flesch-Kincaid Grade Level (FKGL), Flesch reading ease (FRE) score, Gunning Fog Index, Dale-Chall score, and automated readability index (ARI), along with the word count and total character length. Lower FKGL, Gunning Fog, Dale-Chall, and ARI scores, together with higher FRE scores, reflect greater ease of comprehension. For each metric, we calculated the mean, SD, and range across all generated questions. These readability indices provided a quantitative benchmark of patient-friendliness and helped identify potential barriers to comprehension in the context of clinical communication. They were further compared with patient survey ratings of perceived understanding and usefulness to triangulate readability with real-world patient perspectives.

#### Evaluation by Patients

Following clinician review, we conducted a patient evaluation study to assess the understandability and perceived usefulness of the LLM-generated questions. Adult participants (aged ≥18 years) were recruited through three channels: (1) the Florida State University Institute for Successful Longevity (ISL) volunteer registry, (2) flyers posted on social media platforms, and (3) the Prolific online survey platform. Eligibility criteria included normal or corrected-to-normal vision; ability to speak and understand English; and a self-reported diagnosis of at least one of the following conditions—type 1 or type 2 diabetes, chronic kidney disease, heart disease or heart failure, hyperlipidemia, hypertension, or liver disease. Recruitment emails were sent to ISL registry members, who could voluntarily enroll via a survey link. Participants recruited through the ISL registry or social media were entered into a draw for 1 of 3 Amazon gift cards worth US $30, while Prolific participants received US $8 for survey completion. After they provided demographic and health information, each participant was presented with a hypothetical patient profile containing laboratory results aligned with their reported conditions. They were then shown 10 selected question items for 1 of the 3 clinical scenarios (scenario 1 [patient #2], scenario 2 [patient #5], and scenario 3 [patient #6]) generated by the LLM prompt in round 3 and asked to rate each question for understandability and usefulness. For each participant, we calculated a participant-level composite understandability score by averaging that participant’s understandability ratings across the 10 questions assigned to their clinical scenario. We similarly calculated a participant-level composite usefulness score by averaging that participant’s usefulness ratings across the same 10 questions. Scenario-level mean (SD) scores were then calculated using these participant-level composite scores, rather than individual item-level ratings, as the unit of analysis.

The questions were selected based on clinician evaluation results, prioritizing questions rated as clinically appropriate and relevant, while also ensuring diversity across question types (eg, laboratory interpretation, medication-related questions, prognosis/next steps, lifestyle guidance, and monitoring). This approach was used to present patients with a representative and clinically meaningful subset of questions while minimizing participant burden. If a participant had diabetes and hyperlipidemia, they were assigned to scenario 3. Otherwise, if a participant had diabetes or liver disease but not hyperlipidemia, they were assigned to scenario 2. Otherwise, if a participant had chronic kidney disease, hypertension, heart disease or heart failure, or hyperlipidemia, they were assigned to scenario 1. This patient-centered evaluation provided critical insight into the accessibility and perceived value of LLM-generated question prompts from the end-user perspective.

## Results

### LLM Prompt Engineering and Clinician-in-the-Loop Review and Refinement

Together across all rounds, a total of 486 questions were generated. In round 1, GPT-4o generated 126 questions across 3 patient profiles, with 34, 44, and 48 questions generated for patients 1 to 3, respectively. In round 2, GPT-4o generated 120 questions across 6 profiles, with 20 questions per profile. In round 3, GPT-4o generated 180 questions across 9 profiles, with 20 questions per profile. In round 3, LLaMA 3.2 was additionally applied to the 3 newly introduced profiles, generating 60 questions. The between-model comparison was restricted to the matched round 3 subset of patients 7 to 9, consisting of 60 GPT-4o questions and 60 LLaMA 3.2 questions.

### Round 1: Initial Prompt Generation and Evaluation

We developed an initial prompt (Textbox S1 in Multimedia Appendix 2) to generate patient-centered questions for structured clinical profiles (based on demographics, laboratory test results, current medications, and documented diagnoses). The prompt instructed the model to (1) generate 4 questions per laboratory test, (2) rank questions by medical urgency and relevance to the patient’s conditions, (3) emphasize actionable next steps for patients, and (4) ensure that the questions could be addressed within a standard 15-minute primary care visit. Using 3 clinical cases, GPT-4o produced 3 sets of 40 to 60 questions. Each set was independently reviewed by a board-certified family physician, who rated the questions on clarity (ie, clear phrasing), clinical validity, clinical appropriateness, significance for the patient’s health, and willingness to answer. Across sets, 96.8% (122/126) of questions were rated as clearly phrased and 90.5% (114/126) as clinically valid. The mean Likert scores were 3.98 (SD 1.26) for clinical appropriateness, 4.08 (SD 1.05) for significance for the patient’s health, and 4.29 (SD 0.85) for willingness to answer. Semistructured interviews with the 3 physicians provided additional insights. Questions addressing abnormal values of key laboratory markers for chronic conditions were viewed as particularly important, as were those directly linked to a patient’s active diagnoses. At the same time, reviewers noted occasional hallucinations, such as questions incorrectly linking insulin use to thyroxine levels, underscoring the need for iterative refinement of prompt design.

### Round 2: Tailoring LLM Outputs to Clinical Context

Building on round 1 findings, we refined the question generation module to improve specificity, reduce redundancy, and enhance clinical relevance. Refinements included (1) limiting coverage to 37 laboratory tests most relevant for older adults; (2) focusing generation on abnormal laboratory values and explicitly linking results to active diagnoses (eg, type 2 diabetes mellitus, chronic kidney disease, and hyperlipidemia) and current medications (eg, insulin detemir and diltiazem); (3) emphasizing actionable and time-sensitive queries, such as medication adjustments or follow-up test recommendations; (4) calibrating language to a sixth-grade reading level to improve accessibility for patients with limited health literacy; and (5) capping output at 20 ranked questions per case to align with the time constraints in a typical 15-minute encounter. These adjustments yielded more targeted, interpretable, and clinically actionable outputs. Textbox S2 in Multimedia Appendix 2 shows the round 2 prompt template.

**Figure 2.**
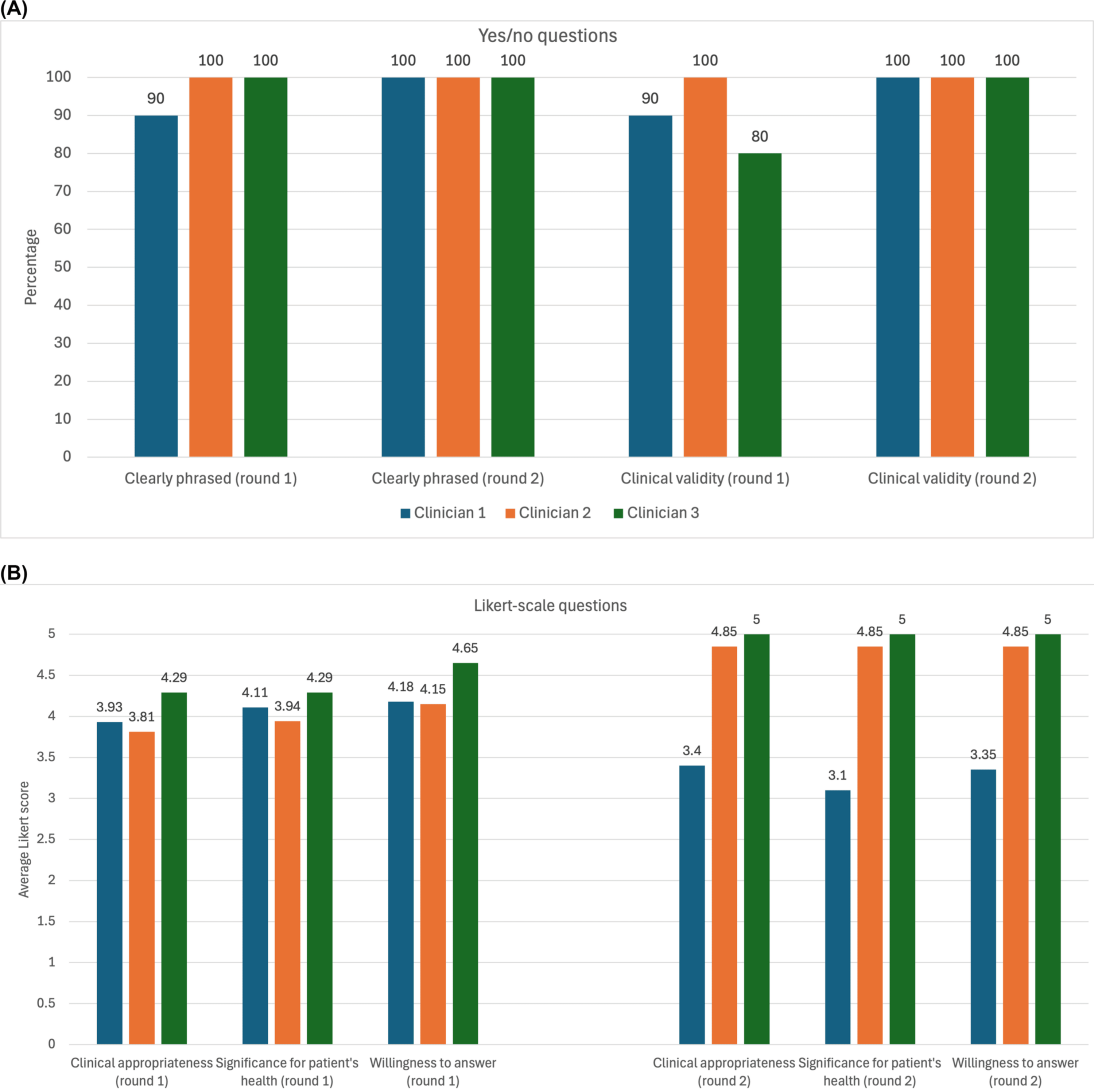
Clinician evaluation of large language model–generated questions across rounds 1 and 2 on (A) two yes or no questions—(1) clear phrasing (ie, clarity) and (2) clinical validity—and (B) three Likert-scale questions: (1) clinical appropriateness (ie, the likelihood of being asked by a patient in primary care setting), (2) significance for the patient’s health, and (3) willingness to answer. This figure summarizes the structured evaluation framework applied to compare outputs across iterative refinement rounds.

Clinician evaluations confirmed substantial improvements over round 1, based on the same 3 patient profiles (#1, #2, #3). All questions (100%, 60/60) were rated as clearly phrased and clinically valid. Mean scores for the 3 Likert-scale questions increased: the scores increased from 3.98 (SD 1.26) to 4.41 (SD 0.98) for clinical appropriateness, from 4.08 (SD 1.05) to 4.32 (SD 1.10) for significance for the patient’s health, and from 4.29 (SD 0.85) to 4.40 (SD 0.85) for willingness to answer. These results suggest that round 2 prompts produced questions that were both clearer and more aligned with patient-provider communication priorities. Figure 2 shows the evaluation results of 3 clinicians on both the binary metrics and Likert-scale metrics for the questions generated in round 1 and round 2. As shown in Figure 2, clinician ratings on Likert-scale measures were generally stable or improved from round 1 to round 2 following prompt refinement. However, a modest decrease in Likert scores was observed for clinician 1 in round 2. This variation likely reflects within-rater variability associated with the approximately 4-week interval between evaluation rounds and the subjective nature of Likert-scale judgments rather than a systematic decline in question quality. Similar variability across clinicians was observed throughout the study, consistent with the low interrater agreement reported.

### Case Study

To illustrate the difference, we examined a White man aged 78 years with multiple chronic conditions, including type 2 diabetes mellitus, chronic kidney disease, and hyperlipidemia. His laboratory profile included elevated triglycerides (329 mg/dL), a creatinine level of 2 mg/dL, and reduced eGFR (37 mL/min/1.73 m^2^). Active medications included multiple insulins, mirabegron, and cefaclor, and diagnoses ranged from urinary tract infection to myelodysplastic syndrome. Table 2 compares the questions generated for this case in round 1 and round 2. Round 2 questions more consistently highlighted abnormal values, linked results to active conditions, and suggested concrete next steps, in contrast to round 1 outputs that were broader and occasionally tangential.

**Table 2.** Comparable questions generated for case 1 from GPT-4o using prompts in round 1 and round 2.

Questions generated by round 1 prompt	Questions generated by round 2 prompt
What symptoms might indicate a worsening of kidney function in light of my GFR of 37 mL/min/1.73 m^2^?	My glomerular filtration rate is 37 mL/min/1.73 m^2^, which seems low. Could this indicate worsening kidney function, and should we adjust any medications, especially with my diabetes and kidney cyst?
Given my creatinine level of 2 mg/dL, should we consider adjusting my medications?	With a creatinine level of 2 mg/dL and reduced kidney function, should I be cautious with my diabetes medications like insulin lispro? Are there alternative dosing strategies?
Considering my history of mixed hyperlipidemia, how should I interpret and manage my triglyceride level of 329 mg/dL?	My triglycerides are very high at 329 mg/dL. How urgent is it to lower this, and what steps should I take, especially with my diagnosis of mixed hyperlipidemia?
What are the implications of my current medication on managing my mixed hyperlipidemia, given my cholesterol level of 185 mg/dL?	My cholesterol is at 185 mg/dL, which I believe is in the normal range, but combined with high triglycerides, is there a risk for heart disease, and should I consider additional lipid-lowering medications?
Are there any dietary or lifestyle changes I can make to improve my chloride level of 107 mmol/L?	My chloride level is at 107 mmol/L. Is this within a normal range, and does it impact my kidney or overall electrolyte balance, especially given my kidney function?
What lifestyle changes can help improve my overall lipid profile given my triglyceride and cholesterol levels?	Given the elevated triglycerides and diabetes, should I be considering any changes to my current diet, exercise routine, or medication?
Considering my mixed hyperlipidemia, how should we manage my cholesterol level of 185 mg/dL to prevent cardiovascular issues?	Should we check my blood pressure more frequently at home, considering my kidney function and high cholesterol, and what range should I aim for?

### Round 3: Final Refinement of LLM Outputs

Building on round 2 results and clinician feedback, we developed a final prompt (Textbox S3 in Multimedia Appendix 2) designed to maximize the clarity, clinical relevance, and consistency of LLM outputs. Key refinements included a few-shot learning approach, in which examples of highly rated and poorly rated questions from previous rounds were embedded directly into the prompt to guide the model toward the desired tone and specificity. In addition, the prompt incorporated structured stepwise instructions, explicit output formatting, and clearer alignment between abnormal laboratory results, medical history, and actionable next steps. These changes shifted the model from relying on implicit expectations to following explicit, clinician-informed rules, thereby improving the consistency and patient-centeredness of generated outputs.

For round 3, three clinical cases were selected in consultation with the family physicians. Using prompt 3, the models generated 20 questions for each case. All 3 physicians independently evaluated every generated question, allowing for the calculation of interrater reliability in addition to descriptive ratings. This final evaluation provided a rigorous assessment of the optimized prompt’s ability to generate clinically coherent, actionable, and patient-friendly questions suitable for supporting real-time communication during primary care visits. Although 20 questions were generated per case for standardized evaluation, a smaller prioritized subset (eg, top 5‐10 questions) is intended for real-world clinical use based on relevance and time constraints.

### Analysis of GPT-4o–Generated Questions in Round 2 vs Round 3

**Figure 3.**
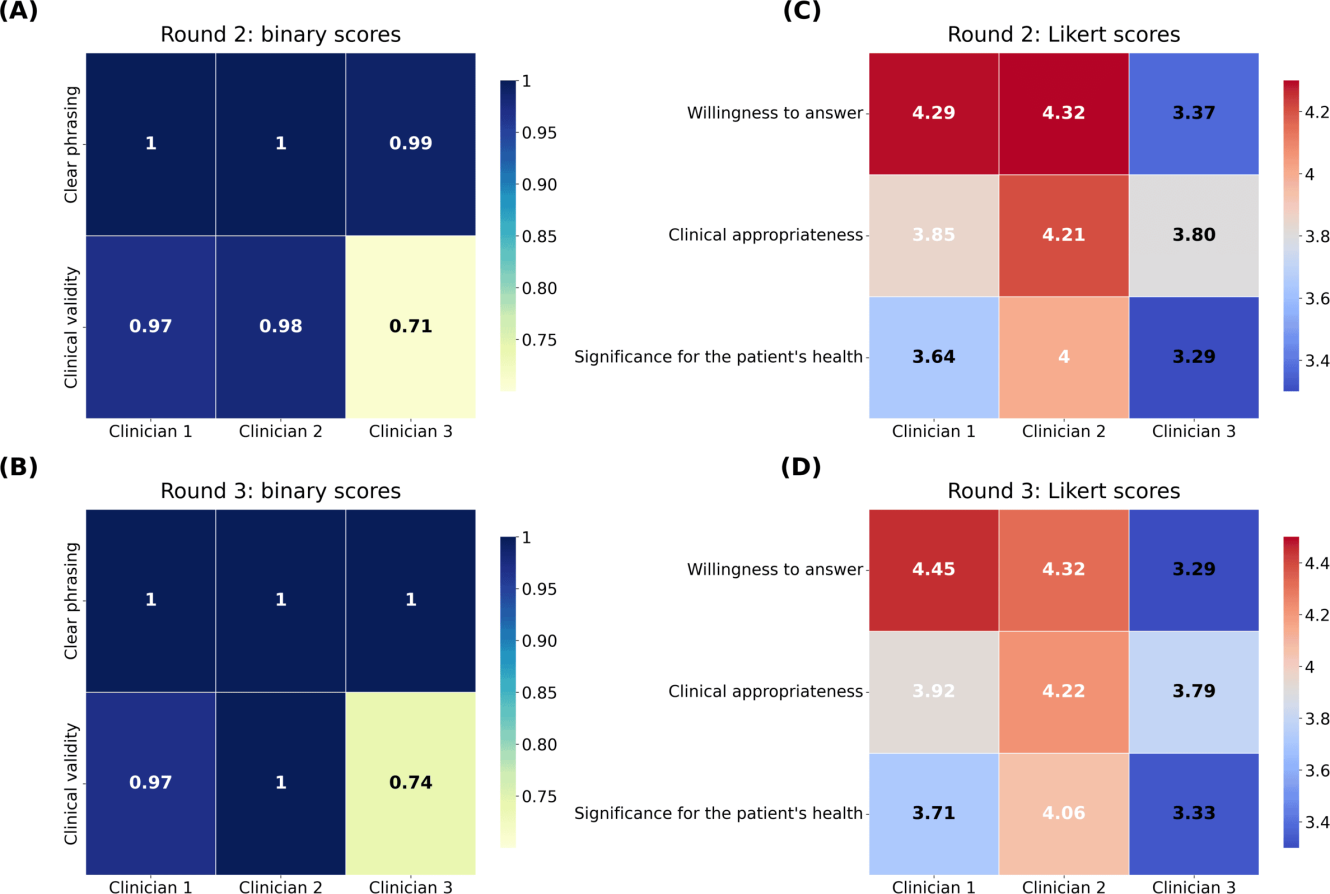
Clinician ratings of GPT-4o–generated questions across rounds 2 and 3. This figure summarizes binary and Likert-scale evaluations of generated questions by 3 family physicians. Panels (A) and (B) show binary scores for clarity of phrasing and clinical validity in rounds 2 and 3, respectively. Panels (C) and (D) display Likert ratings across three dimensions: (1) clinical appropriateness, (2) significance for the patient’s health, and (3) willingness to answer. Scores are presented as heat maps, with darker colors indicating higher values.

Figure 3 illustrates clinician evaluations of GPT-4o–generated questions across rounds 2 and 3 using both binary (yes/no) and Likert-scale metrics. For the binary criteria—*clear phrasing* and *clinical validity*—each clinician reviewed 20 questions per round. A maximum score of 20 indicates that the clinician answered “yes” for all 20 questions. As shown in panels A and B, the clinicians consistently rated the questions as clearly phrased, with scores close to 100% in both rounds. However, there was greater variation in the *clinical validity* ratings, particularly from clinician 3, who gave a noticeably lower score (71% in round 2 and 74% in round 3), suggesting that she found fewer questions to be clinically meaningful. Panels C and D present Likert-scale evaluations (range 1‐5) across 3 dimensions: willingness to answer, clinical appropriateness, and its significance for the patient’s health. In round 2 (panel C), clinicians 1 and 2 rated the questions favorably (mostly above 4), whereas clinician 3 gave consistently lower scores, especially for *willingness to answer* (3.37) and *significance for the patient’s health* (3.29). In round 3 (panel D), following prompt refinements, average scores given by clinicians 1 and 2 improved, with clinician 2 giving the highest score of 4.45 for *willingness to answer*. Despite these improvements, clinician 3 maintained more critical ratings across all criteria. Overall, the figure highlights strong agreement on language clarity but variation in clinical interpretation, emphasizing the need for multiclinician feedback and continued prompt optimization. Additionally, Figure S1 in Multimedia Appendix 2 presents the average clinician ratings of LLM-generated questions across key evaluation criteria, aggregated from rounds 2 and 3.

Clinician evaluations of LLM-generated clinical questions in round 3 were visualized as heat maps across both Likert and binary rating scales (Figures S2-S6 in Multimedia Appendix 2). Likert-based heat maps captured ratings for clinical appropriateness (Figure S2 in Multimedia Appendix 2), significance for the patient’s health (Figure S3 in Multimedia Appendix 2), and willingness to answer (Figure S4 in Multimedia Appendix 2), enabling a direct comparison between GPT-4o and LLaMA 3.2 across the 1 to 5 scale. Binary heat maps further illustrated clinician judgments on whether questions were clearly phrased (Figure S5 in Multimedia Appendix 2) and whether they demonstrated clinical validity (Figure S6 in Multimedia Appendix 2), highlighting patterns of clarity and appropriateness in the model outputs.

Tables3  4 summarize the clinicians’ evaluations of GPT-4o–generated laboratory test questions across rounds 2 and 3 using binary and Likert-scale scores. The binary ratings show that the questions were consistently clear for all clinicians (mean 1, SD 0), while the ratings for clinical validity varied, especially for clinician 3. The Likert scores were generally favorable, with clinicians 1 and 2 giving higher ratings than clinician 3 across the dimensions clinical appropriateness, significance for the patient’s health, and willingness to answer. Table 5 shows the interrater agreement (estimated by the ICC) between 3 clinicians or 2 clinicians (excluding the outlier). The ICC for the 3 clinicians signified low interrater agreement (ICC <0.3) across all criteria, indicating variability in clinician judgment despite consistent clarity in question phrasing. These results highlight that while GPT-4o–generated questions were reliably well phrased, the perceptions of their clinical appropriateness and usefulness differed significantly among clinicians. The ICC for the 2 clinicians who showed higher agreement was noticeably higher, with ICC >0.3 across 2 Likert scores in both round 2 and round 3. Nonetheless, the lack of statistically significant differences between rounds 2 and 3 suggests that prompt refinements had limited measurable impact, although slight trends toward improved ratings—particularly in willingness to answer—were observed. Interrater reliability among clinicians was generally poor, with several negative ICC values observed across both rounds, indicating systematic disagreement. Even after excluding the lowest-rating clinician, interrater agreement improved only modestly (ICC range: 0.121‐0.557), remaining in the poor to fair range. For these specific metrics, the confidence intervals included zero and therefore did not support a claim of agreement above chance. The low interrater reliability underscored the need for clearer evaluation guidelines and potentially a larger panel of reviewers to capture diverse clinical perspectives when assessing AI-generated medical content.

**Table 3.** Mean clinician evaluation scores on binary metrics for GPT-4o–generated laboratory test questions in rounds 2 and 3 (n=120)^a^.

Binary metric	Clinician rating on binary questions, mean (SD)		
	Round 2 questions	Round 3 questions	*P* value^b^
Clear phrasing			
Clinician 1	1 (0)	1 (0)	>.99
Clinician 2	1 (0)	1 (0)	>.99
Clinician 3	0.99 (0.09)	1 (0)	.32
Clinical validity			
Clinician 1	0.97 (0.18)	0.97 (0.16)	.70
Clinician 2	0.98 (0.13)	1 (0)	.16
Clinician 3	0.71 (0.46)	0.74 (0.44)	.57

a Binary ratings (number of questions=20 per patient clinical profile).

b Comparisons between round 2 and round 3 were performed using the Mann-Whitney *U* test. Bonferroni correction was applied for the 2 scores: clear phrasing and clinical validity; the adjusted significance threshold was *P*=.025. Exact *P* values are reported.

**Table 4.** Mean clinician evaluation scores on Likert-scale metrics for GPT-4o–generated laboratory test questions in rounds 2 and 3 (n=120)^a^.

Scale	Clinician rating on Likert-scale questions, mean (SD)		
	Round 2 questions	Round 3 questions	*P* value^b^
Clinical appropriateness			
Clinician 1	3.85 (0.72)	3.93 (0.71)	.46
Clinician 2	4.21 (0.70)	4.22 (0.76)	.80
Clinician 3	3.80 (1.24)	3.79 (1.22)	.92
Significance for the patient’s health			
Clinician 1	3.64 (0.78)	3.73 (0.77)	.48
Clinician 2	4 (0.72)	4.06 (0.77)	.54
Clinician 3	3.29 (1.39)	3.33 (1.26)	.85
Willingness to answer			
Clinician 1	4.29 (0.81)	4.46 (0.73)	.098
Clinician 2	4.32 (0.66)	4.32 (0.69)	.94
Clinician 3	3.37 (1.45)	3.29 (1.43)	.66

a Scores are based on a 5-point Likert scale (number of questions=20 per patient clinical profile) evaluating the following dimensions: clinical appropriateness (1=very unlikely, 2=unlikely, 3=neutral, 4=likely, 5=very likely), significance for the patient’s health (1=very insignificant, 2=insignificant, 3=neutral, 4=significant, 5=very significant), and willingness to answer the question (1=would not answer, 2=reluctantly answer, 3=neutral, 4=willing to answer, 5=very willing to answer).

b Comparisons between round 2 and round 3 were performed using the Mann-Whitney *U* test. Bonferroni correction was applied across the 3 Likert-scale scores; the adjusted significance threshold was *P*=.0167. Exact *P* values are reported.

**Table 5.** Intraclass correlation coefficients (ICCs) for the 3 Likert-scale metrics among the clinicians.

	ICC (95% CI)
Intraclass correlation for the 3 metrics among all the 3 clinicians	
Round 2	
Clinical appropriateness	−0.06 (−0.37 to 0.60)
Significance for the patient’s health	0.11 (−0.29 to 0.73)
Willingness to answer	−0.30 (−0.45 to 0.25)
Round 3	
Clinical appropriateness	−0.03 (−0.35 to 0.63)
Significance for the patient’s health	0.30 (−0.18 to 0.82)
Willingness to answer	−0.11 (−0.38 to 0.55)
Intraclass correlation for the 3 metrics among 2 clinicians with higher ratings	
Round 2	
Clinical appropriateness	−0.29 (−0.86 to 0.60)
Significance for the patient’s health	0.33 (−0.56 to 0.87)
Willingness to answer	0.41 (−0.50 to 0.89)
Round 3	
Clinical appropriateness	0.12 (−0.70 to 0.80)
Significance for the patient’s health	0.56 (−0.34 to 0.92)
Willingness to answer	0.51 (−0.40 to 0.91)

### Comparative Analysis of GPT-4o– and LLaMA 3.2–Generated Questions in Round 3

**Figure 4.**
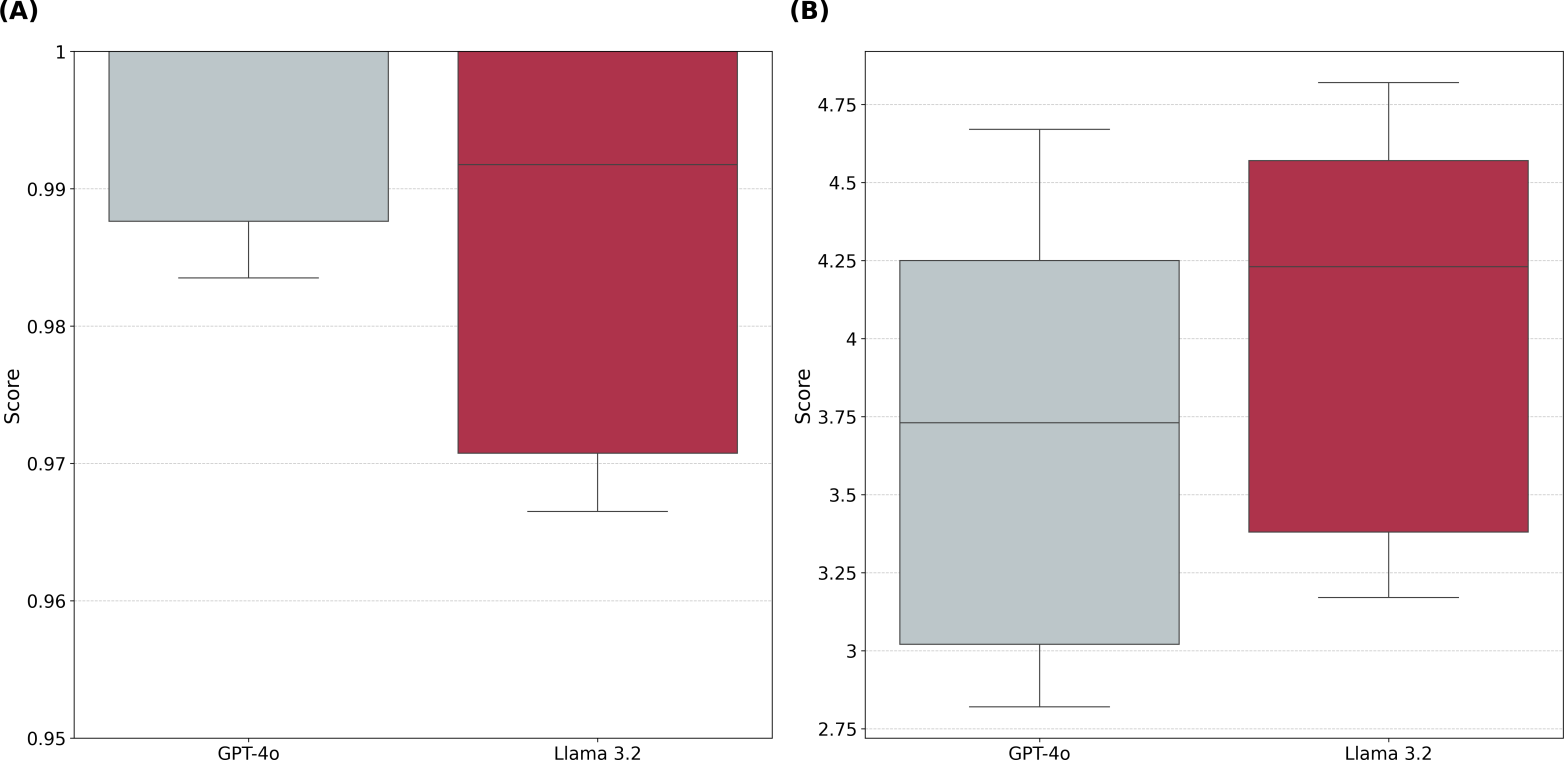
Comparison of large language model (LLM) performance in round 3 using clinician scores. (A) Binary score distribution shows that both GPT-4o and LLaMA 3.2 achieved high accuracy near 1, with GPT-4o displaying slightly less variability; no significant difference was detected (Mann-Whitney *U* test, *P*=.59). (B) Likert score distribution (1‐5 scale) indicates median ratings of~3.75 for GPT-4o and ~4.25 for LLaMA 3.2, with overlapping variability and no significant difference (*P*=.25). In both panels, boxes represent the IQR, center lines represent medians, and whiskers extend to values within 1.5× IQR.

Figure 4 compares the performance of 2 LLMs—GPT-4o and LLaMA 3.2—based on clinician-assigned scores in round 3. Panel A displays the distribution of binary scores (covering the *clear phrasing* and *clinical validity* criteria), while panel B shows the distribution of Likert-scale scores (evaluating *clinical appropriateness*, *significance for the patient’s health*, and *willingness to answer*). In both panels, box plots visualize the median, IQR, and score dispersion for each model. For the binary ratings, both models performed similarly, with median scores near the maximum value: GPT-4o had a median score of 1 (IQR 0.99-1), and LLaMA 3.2 had a median score of 0.99 (IQR 0.97-1). The Mann-Whitney *U* test showed overlapping binary score distributions between GPT-4o and LLaMA 3.2 (*P*=.59). The Likert-scale scores also showed overlapping distributions across the 2 models, with median ratings of 3.73 (IQR 3.02-4.25) for GPT-4o and 4.23 (IQR 3.38-4.57) for LLaMA 3.2 (*P*=.25). These findings suggest comparable clinician ratings for GPT-4o and LLaMA 3.2 in round 3 when the binary and Likert-scale ratings were pooled across evaluation dimensions. Overall, this analysis demonstrates that GPT-4o and LLaMA 3.2 performed comparably in generating clinically meaningful, clearly phrased questions, based on evaluations from practicing clinicians in round 3.

Box plots show the distribution of clinician-assigned binary scores (*clear phrasing* and *clinical validity*) and Likert-scale scores (*clinical appropriateness*, *significance for the patient’s health*, and *willingness to answer*) for questions generated by GPT-4o and LLaMA 3.2 in round 3. Median scores, IQRs, and outliers are visualized. Mann-Whitney *U* tests indicated no statistically significant difference between the models (*P*=.59 for binary scores; *P*=.25 for Likert scores), suggesting comparable performance across both evaluation types.

**Figure 5.**
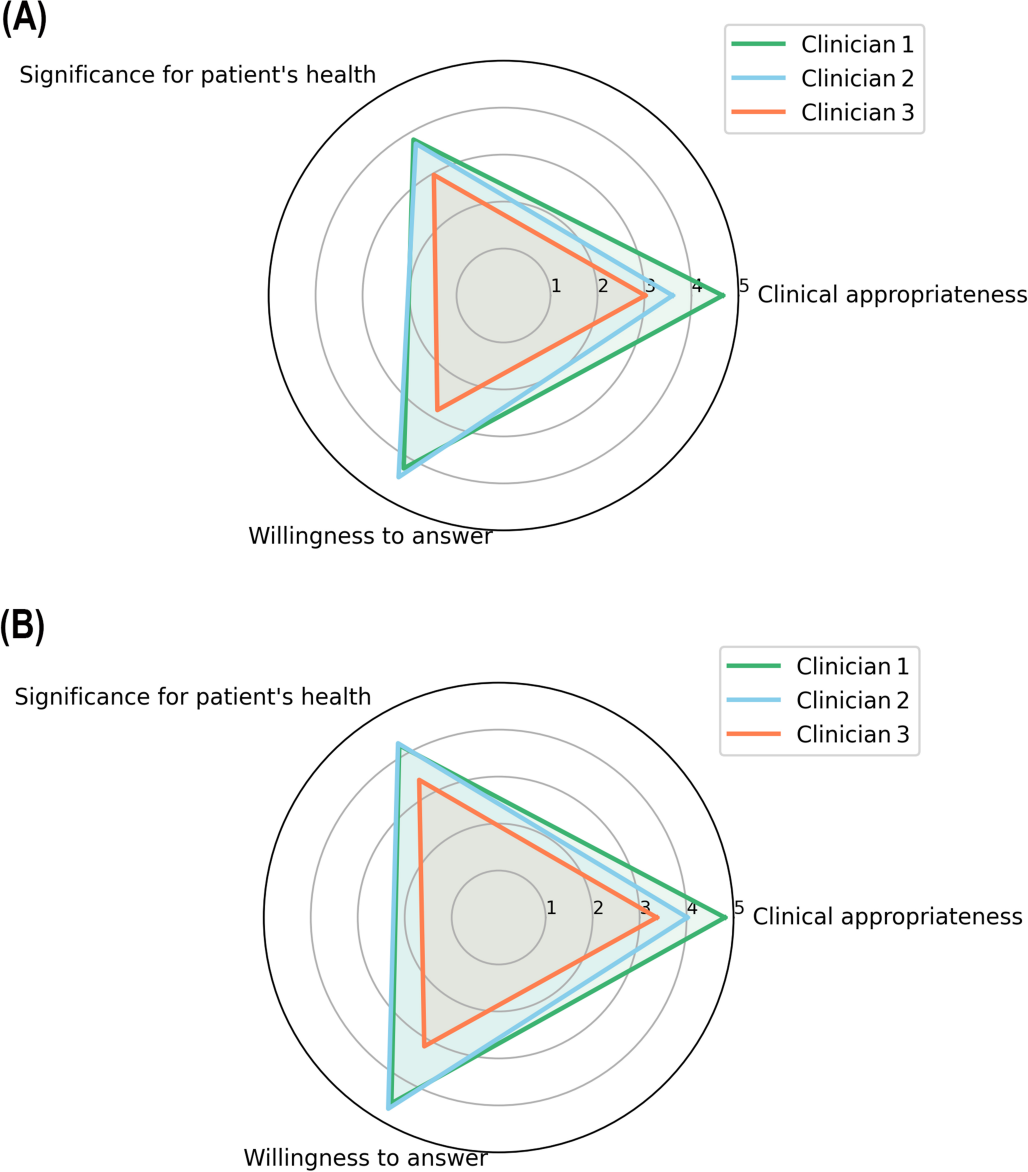
Clinician Likert score profiles for laboratory test–related questions generated by 2 large language models in round 3. (A) GPT-4o questions and (B) LLaMA 3.2 questions. Axes show the 3 evaluation criteria—clinical appropriateness, significance, and clinician willingness—rated on a 1 to 5 scale (higher is better). Shaded polygons represent the mean scores from clinician 1 (medium sea green), clinician 2 (sky blue), and clinician 3 (coral).

Figure 5 compares the clinicians’ Likert score profiles for GPT-4o–generated (panel A) and LLaMA 3.2–generated (panel B) laboratory test questions in round 3. The 3 axes represent primary care relevance, significance, and clinician willingness, each scored from 1 (lowest) to 5 (highest). Across both models, clinician 1 and clinician 2 generally rated the questions higher than clinician 3 did, as shown by their larger polygons. GPT-4o received slightly higher or comparable ratings on all 3 dimensions than LLaMA 3.2, especially in primary care relevance. The radar plots visually highlight interclinician variation, with clinician 3 consistently providing more conservative scores.

Table 6 compares clinicians’ evaluations of questions generated by GPT-4o and LLaMA 3.2, using both binary and Likert-scale scores. For the round 3 between-model comparison, analyses were restricted to the 3 overlapping clinical profiles—patients 7, 8, and 9—for which both GPT-4o and LLaMA 3.2 generated questions. Each model generated 20 questions per profile, resulting in a matched comparison set of 60 questions per model. GPT-4o–generated questions from patients 1 to 6 were excluded from the between-model comparison to avoid case-mix confounding. In the binary evaluation (Table 6), all 3 clinicians unanimously rated the questions from both models as clearly phrased with perfect scores of 1 (SD 0), showing strong agreement in language clarity. For clinical validity, ratings were more variable. Clinician 1 rated GPT-4o and LLaMA 3.2 similarly, with mean scores of 0.98 (SD 0.13) and 1 (SD 0), respectively (*P*=.33). Clinician 2 also rated both models highly, with mean scores of 1 (SD 0) for GPT-4o and 0.97 (SD 0.18) for LLaMA 3.2 (*P*=.16). Clinician 3 gave lower clinical validity ratings overall, with GPT-4o rated 0.61 (SD 0.49) compared with 0.43 (SD 0.50) for LLaMA 3.2 (*P*=.055). In Table 6, the binary metric *clear phrasing* reached a ceiling effect (mean score 1, SD 0, across all clinicians for both models in round 3), limiting its ability to differentiate model performance. This suggests that the metric lacks sensitivity to capture subtle or nuanced differences in clarity between GPT-4o and LLaMA 3.2 outputs. Future evaluations may benefit from more granular or scaled measures to better distinguish variations in clarity. Table 7 shows Likert-scale scores (1-5) assessing clinical appropriateness, significance for the patient’s health, and willingness to answer. Across all 3 clinicians, LLaMA 3.2–generated questions showed higher mean Likert scores than GPT-4o–generated questions across several dimensions. However, after applying the Bonferroni-adjusted significance threshold of *P*=.0167, only selected comparisons remained statistically significant.

**Table 6.** Round 3 clinician evaluation results on binary metrics for large language model–generated laboratory test questions (n=60).^a^

Binary question	Clinician score, mean (SD)		
	GPT-4o	LLaMA 3.2	*P* value^b^
Clear phrasing			
Clinician 1	1 (0)	1 (0)	>.99
Clinician 2	1 (0)	1 (0)	>.99
Clinician 3	1 (0)	1 (0)	>.99
Clinical validity			
Clinician 1	0.98 (0.13)	1 (0)	.33
Clinician 2	1 (0)	0.97 (0.18)	.16
Clinician 3	0.61 (0.49)	0.43 (0.50)	.06

a Binary ratings (number of questions=20 per patient clinical profile).

b Comparisons between GPT-4o and LLaMA 3.2 were performed using the Mann-Whitney *U* test. Bonferroni correction was applied for the 2 scores—clear phrasing and clinical validity—the adjusted significance threshold was *P*=.025. Exact *P* values are reported. The round 3 between-model comparison was restricted to the matched subset of patients 7‐9, for which both GPT-4o and LLaMA 3.2 generated 20 questions per patient clinical profile, yielding 60 questions per model.

**Table 7.** Round 3 clinician evaluation results on Likert-scale metrics for large language model–generated laboratory test questions (n=60).^a^

Likert-scale question	Clinician score, mean (SD)		
	GPT-4o	LLaMA 3.2	*P* value^b^
Clinical appropriateness			
Clinician 1	3.60 (0.76)	4.02 (0.68)	.002
Clinician 2	4.67 (0.60)	4.82 (0.62)	.04
Clinician 3	3.02 (0.85)	3.37 (0.78)	.017
Significance for the patient’s health			
Clinician 1	3.73 (0.82)	4.28 (0.74)	<.001
Clinician 2	3.83 (0.74)	4.23 (0.79)	.004
Clinician 3	2.97 (0.88)	3.38 (0.72)	.005
Willingness to answer			
Clinician 1	4.47 (0.89)	4.70 (0.62)	.17
Clinician 2	4.25 (0.54)	4.57 (0.81)	<.001
Clinician 3	2.82 (0.98)	3.17 (0.85)	.04

a Scores are based on a 5-point Likert scale (number of questions=20 per patient clinical profile) evaluating the following dimensions: clinical appropriateness (1=very unlikely, 2=unlikely, 3=neutral, 4=likely, 5=very likely), significance for the patient’s health (1=very insignificant, 2=insignificant, 3=neutral, 4=significant, 5=very significant), and willingness to answer the question (1=would not answer, 2=reluctantly answer, 3=neutral, 4=willing to answer, 5=very willing to answer).

b Comparisons between GPT-4o and LLaMA 3.2 were performed using the Mann-Whitney *U* test. Bonferroni correction was applied across the 3 Likert-scale scores; the adjusted significance threshold was *P*=.0167. Exact *P* values are reported. The round 3 between-model comparison was restricted to the matched subset of patients 7‐9, for which both GPT-4o and LLaMA 3.2 generated 20 questions per patient clinical profile, yielding 60 questions per model.

For clinical appropriateness, LLaMA 3.2 received higher mean scores than GPT-4o from clinician 1 (mean 4.02, SD 0.68 vs mean 3.60, SD 0.76; *P*=.002), clinician 2 (mean 4.82, SD 0.62 vs mean 4.67, SD 0.60; *P*=.042), and clinician 3 (mean 3.37, SD 0.78 vs mean 3.02, SD 0.85; *P*=.017). Only the comparison for clinician 1 met the Bonferroni-adjusted significance threshold. The comparisons for clinician 2 and clinician 3 did not meet the adjusted threshold and should therefore be interpreted descriptively.

For significance for the patient’s health, LLaMA 3.2 received significantly higher scores than GPT-4o from clinician 1 (mean 4.28, SD 0.74 vs mean 3.73, SD 0.82; *P*<.001), clinician 2 (mean 4.23, SD 0.79 vs mean 3.83, SD 0.74; *P*=.004), and clinician 3 (mean 3.38, SD 0.72 vs mean 2.97, SD 0.88; *P*=.005), all of which met the Bonferroni-adjusted significance threshold. For willingness to answer, LLaMA 3.2 received higher scores than GPT-4o from clinician 2 (mean 4.57, SD 0.81 vs mean 4.25, SD 0.54; *P*<.001), which met the Bonferroni-adjusted significance threshold. The difference for clinician 3 (mean 3.17, SD 0.85 vs mean 2.82, SD 0.98; *P*=.038) did not meet the Bonferroni-adjusted significance threshold and should be interpreted descriptively. The difference for clinician 1 was smaller and not statistically significant (mean 4.70, SD 0.62 vs mean 4.47, SD 0.89; *P*=.17).

Although LLaMA 3.2 showed comparable performance on select metrics, its lower ratings on clinical validity by some clinicians indicate that a significant proportion of generated questions may lack clinical validity. This variability underscores potential safety risks and the necessity of clinician-guided use. Table 8 reports interrater agreement using ICC for each model across the 3 Likert-scale criteria. For GPT-4o, *significance for the patient’s health* showed the highest interrater reliability among the 3 Likert-scale metrics, with an ICC of 0.881. For LLaMA 3.2, *clinical appropriateness* showed the highest interrater reliability, with an ICC of 0.886. However, ICC values were not consistently high across all domains, and confidence intervals remained wide, limiting interpretability. These results indicate that while LLaMA may have produced questions seen as more clinically relevant, variability in clinician interpretation remains, highlighting the importance of multiexpert validation. While higher ICC values were observed for specific metrics and models (eg, GPT-4o and LLaMA 3.2 in round 3), these were not consistent across all domains, and confidence intervals remained wide, limiting interpretability.

**Table 8.** Intraclass correlation coefficients (ICCs) for the 3 metrics among the 3 clinicians in round 3.

Large language model and metric	ICC (95% CI)
GPT-4o	
Clinical appropriateness	0.31 (–0.35 to 0.97)
Significance for the patient’s health	0.88 (0.28 to 1)
Willingness to answer	0.29 (–0.36 to 0.97)
LLaMA 3.2	
Clinical appropriateness	0.89 (0.30 to 1)
Significance for the patient’s health	0.49 (–0.27 to 0.98)
Willingness to answer	0.47 (–0.28 to 0.98)

### Readability Assessment

Table 9 compares the readability and length of clinical questions generated by GPT-4o and LLaMA 3.2 using standard readability indices. These metrics help assess how easily patients can understand the model-generated questions. For each language model, we report the mean (SD) and range (min-max) across 7 metrics: FKGL, FRE score, Gunning Fog Index, Dale-Chall score, ARI, number of words per question, and total character-based question length. Lower grade levels (FKGL, Gunning Fog, Dale-Chall, and ARI) and higher FRE scores indicate greater readability. These metrics help assess how easily patients can understand the model-generated questions.

**Table 9.** Readability metrics and length characteristics of large language model (LLM)–generated clinical questions in round 3.

LLM and readability score/words	Mean (SD)	Range (min to max)
GPT-4o		
Flesch-Kincaid Grade Level	6.79 (1.83)	2.90 to 10.30
Flesch reading ease score	66.05 (13.02)	37.47 to 94.15
Gunning Fog Index	9.01 (2.36)	4.20 to 14.80
Dale-Chall score	11.29 (1.21)	8.68 to 13.73
Automated readability index	7.06 (1.93)	2.60 to 12.10
Number of words	23.75 (4.76)	16 to 36
Question length (number of characters)	138.65 (26.96)	92 to 217
LLaMA 3.2		
Flesch-Kincaid Grade Level	9.34 (3.47)	3.50 to 17.80
Flesch reading ease score	56.58 (16.67)	17.68 to 81.80
Gunning Fog Index	11.53 (3.57)	6.56 to 21.20
Dale-Chall score	12.03 (1.42)	8.87 to 15.19
Automated readability index	10.64 (3.77)	4.80 to 20.20
Number of words	28.55 (5.32)	16 to 39
Question length (number of characters)	173.22 (34.05)	101 to 250

The readability analysis revealed that GPT-4o generated simpler and more concise questions than LLaMA 3.2. GPT-4o–generated questions averaged an FKGL of 6.79, indicating that they were suitable for a sixth-grade to seventh-grade reading level, and an FRE score of 66.05, suggesting fairly easy comprehension. In contrast, LLaMA 3.2 generated more complex language, with a higher FKGL of 9.34 and a lower FRE score of 56.58, indicating the need for a higher literacy level. Additionally, the LLaMA-generated questions were longer on average than the GPT-4o–generated questions—both in word count (mean 28.55, SD 5.32 vs mean 23.75, SD 4.76) and character count (mean 173.22, SD 34.05 vs mean 138.65, SD 26.96)—potentially increasing the cognitive load for patients. These differences suggest that GPT-4o may be more suitable for patient-facing applications where readability is critical.

Multimedia Appendix 3 contains the evaluation of LLM-generated clinical laboratory test questions in round 3 reviewed by clinicians, with each entry organized by question ID, laboratory test, patient context, LLM, and clinician identifier, along with standardized evaluation metrics. For instance, Q41 presents a GPT-4o–generated question addressing an abnormal creatinine level: “My creatinine level is very high at 4.81 mg/dL, which suggests kidney problems. Given my diabetes, hypertension, and use of diltiazem and furosemide, should we adjust my medications or refer me to a kidney specialist?” This question was unanimously rated as clearly phrased (“Yes”) and clinically valid (“Yes”) by all 3 clinicians and received high Likert-scale ratings across the evaluation domains. Q81 shows another GPT-4o–generated question addressing abnormal glucose and HbA_1c_ levels: “My glucose level is very high at 275 mg/dL, and my HbA_1c_ is 8.6%. Given my Type 2 diabetes, do I need to change my diabetes medications or insulin regimen to better control my blood sugar?” This question was also unanimously rated as clearly phrased (“Yes”) and clinically valid (“Yes”) by all 3 clinicians. It achieved high Likert-scale ratings for relevance and actionability: two clinicians gave it perfect scores (5/5) across clinical appropriateness, significance for the patient’s health, and willingness to answer, while the third clinician rated it highly (4/5) across all 3 domains. Overall, each question in the dataset was assessed using 2 binary judgments (clarity and clinical validity) and 3 Likert-scale ratings (clinical appropriateness, significance for the patient’s health, and clinician willingness to answer), providing transparency into the evaluation process and enabling reproducibility while capturing nuanced clinician perspectives on the validity and relevance of LLM-generated clinical questions.

### Patient Evaluation Results

Following clinician review and readability assessment, we conducted a patient-centered evaluation of a subset of the tailored LLM-generated question prompts with 134 patients. Their basic characteristics are shown in Table 10. Each participant rated 10 tailored questions for discussion in a follow-up medical visit for their perceived understandability and usefulness associated with their assigned scenario using 5-point scales (1=strongly disagree, 5=strongly agree). Composite scores were calculated from each of the 10 perceived understandability questions and the 10 usefulness questions (Table 11). Multimedia Appendix 4 provides the item-level question evaluation scores given by patients and the corresponding tailored questions for the clinical scenarios.

**Table 10.** Sample characteristics for patient evaluation (N=134).

Variable	Value
Age (years), mean (SD)	53.6 (16.1)
Sex, n (%)	
Male	41 (30.6)
Female	91 (67.9)
Nonbinary	2 (1.5)
Race, n (%)	
American Indian/Alaska Native	2 (1.5)
Asian	5 (3.7)
Black or African American	19 (14.2)
Native Hawaiian/Pacific Islander	0 (0)
White	104 (77.6)
Multiracial	4 (3)
Hispanic/Latino	13 (9.7)
English proficiency, n (%)	
None or low	6 (4.4)
Moderate	18 (13.4)
Very high	39 (29.1)
Extremely high	71 (53)
Marital status, n (%)	
Single	24 (17.9)
Married	67 (50)
Divorced	21 (15.7)
Separated	1 (0.7)
Widowed	9 (6.7)
Living with partner	12 (9)
Education, n (%)	
Less than high school	1 (0.7)
High school graduate	13 (9.7)
Some college	35 (26.1)
College graduate	51 (38.1)
Master’s degree	26 (19.4)
Doctoral/professional degree	8 (6)
Annual household income (US $), n (%)	
<10,000	8 (6)
10,000‐19,999	8 (6)
20,000‐34,999	12 (9)
35,000‐49,999	22 (16.4)
50,000‐74,999	21 (15.7)
75,000‐99,999	19 (14.2)
100,000‐149,999	25 (18.7)
≥150,000	13 (9.7)
Prefer not to answer	6 (4.5)
Employment, n (%)	
Employed, full time	45 (33.6)
Employed, part time	15 (11.2)
Unemployed (looking)	10 (7.5)
Unemployed (not looking)	3 (2.2)
Retired	41 (30.6)
Student	1 (0.7)
Disabled	6 (4.5)
Self-employed, full time	7 (5.2)
Self-employed part time	2 (1.5)
Unable to work	4 (3)
Health insurance coverage, n (%)	123 (91.8)
Condition group, n (%)	
Hyperlipidemia and diabetes	3 (2.2)
Diabetes or liver disease	52 (38.8)
CVD^a^ or CKD^b^	79 (59)

a CVD: cardiovascular disease.

b CKD: chronic kidney disease.

**Table 11.** Composite understandability and usefulness scores by patient scenario^a^.

Clinical scenario	Patient profile	Participant sample size, n	Composite understandability score, mean (SD)	Composite usefulness score, mean (SD)
Scenario 1: chronic kidney disease or cardiovascular diseases	Patient #2	79	4.12 (0.88)	3.97 (0.86)
Scenario 2: diabetes or liver disease but no hyperlipidemia	Patient #5	52	3.48 (1)	3.48 (0.87)
Scenario 3: hyperlipidemia and diabetes	Patient #6	3	4.50 (0.56)	3.87 (0.99)

a Composite scores were calculated at the participant level. Each participant rated 10 questions for understandability and 10 questions for usefulness within their assigned clinical scenario. The 10 ratings were averaged separately to generate one composite understandability score and one composite usefulness score per participant. The scenario-level means and SDs reported in this table were calculated from these participant-level composite scores.

### Overall Understandability and Perceived Usefulness of Tailored Questions

Across all 30 tailored questions generated, participants generally rated the questions as understandable, while the usefulness ratings were more variable. As shown in Table S1 in Multimedia Appendix 2, 17 of the 30 questions had mean understandability scores of 4 or higher, 7 were in the moderate range between 3.5 and 3.9, and only 6 questions fell below 3.5. Usefulness ratings showed a different distribution: only 9 questions had mean scores of 4 or higher, 11 were in the moderate range, and 10 were rated below 3.5. Thus, while most questions were viewed as at least moderately understandable, fewer were consistently regarded as useful for prompting patient-clinician conversations.

### Average Ratings of Tailored Questions by Clinical Scenarios

Scenario-level composite scores are summarized in Table 11. These values represent participant-level composite scores calculated by averaging each participant’s ratings across the 10 questions assigned to their clinical scenario, separately for understandability and usefulness. Scenario 1 received high composite ratings, with an average score of 4.12 (SD 0.88) for understandability and 3.97 (SD 0.86) for usefulness. None of the questions in scenario 1 had average ratings below 3.5 for either outcome (see Table S1 in Multimedia Appendix 2).

Scenario 3 showed the highest composite understandability score, with an average of 4.50 (SD 0.56), and an intermediate composite usefulness score of 3.87 (SD 0.99). However, these findings should be interpreted cautiously because only 3 participants were assigned to scenario 3. Scenario 2 received the lowest composite ratings, with average scores of 3.48 (SD 1) for understandability and 3.48 (SD 0.87) for usefulness.

Variation in item-level ratings also differed across scenarios (see Table S2 in Multimedia Appendix 2). Scenario 1 showed relatively consistent ratings across the 10 questions, with item-level understandability means ranging from 3.72 (SD 1.25) to 4.46 (SD 0.96) and usefulness means ranging from 3.58 (SD 1.36) to 4.34 (SD 0.83). Scenario 2 showed lower item-level ratings overall, with understandability means ranging from 3.17 (SD 1.38) to 4 (SD 1.05) and usefulness means ranging from 3.06 (SD 1.36) to 3.92 (SD 1.13), suggesting that participants perceived these questions as less understandable and less useful than those in scenario 1. Scenario 3 showed generally high item-level understandability and usefulness ratings but with greater variability for several items. These scenario 3 item-level findings should be interpreted cautiously because only 3 participants were assigned to this scenario.

### Item-Level Variation in Ratings Across Scenarios

At the item level (see Table S2 in Multimedia Appendix 2 and Multimedia Appendix 4), scenario 1 provided the most stable evidence of favorable ratings because it had the largest participant sample size and consistently high item-level scores. For example, the question “Are there any warning signs of worsening kidney function that I should watch for at home, like swelling or changes in urination?” received high ratings for both understandability (mean 4.46, SD 0.96) and usefulness (mean 4.34, SD 0.83). Similarly, “Are there any dietary changes, such as reducing protein or salt, that could help improve my kidney function given my high creatinine levels?” was rated highly for understandability (mean 4.41, SD 0.95) and usefulness (mean 4.20, SD 0.94). Although scenario 3 showed high understandability for some items, such as the estimated average glucose question (mean 5, SD 0), these findings should be interpreted cautiously because only 3 participants rated this scenario. Overall, scenario 1 offered the most reliable example of consistently favorable item-level ratings across both understandability and usefulness.

### Ratings of Tailored Questions by Category

To further examine whether differences in ratings were tied to the type of question rather than the clinical scenario alone, each item was coded into a primary category based on its dominant intent. The categories included laboratory interpretation, prognosis or next steps, medication safety, medication adjustment, lifestyle and self-care, condition impact, and tracking or monitoring. Items that addressed more than one intent were also assigned a secondary code, although averages are reported by primary category. The purpose of this analysis was to explore whether patients responded more favorably to certain kinds of questions, providing insight into how LLM-generated prompts might be refined to align with patient needs.

Category-level averages are presented in Table S3 in Multimedia Appendix 2. Lifestyle and self-care questions received some of the highest ratings (mean understandability score 4.05, SD 0.33; mean usefulness score 3.77, SD 0.38), along with prognosis or next steps (mean understandability score 4.01, SD 0.55; mean usefulness score 3.76, SD 0.53). In contrast, medication safety (mean understandability score 3.79, SD 0.49; mean usefulness score 3.52, SD 0.40) and laboratory interpretation questions (mean understandability score 3.68, SD 0.28; mean usefulness score 3.67, SD 0.65) were rated lower on average. Medication adjustment questions showed moderate means (mean understandability score 3.96, SD 0.43; mean usefulness score 3.75, SD 0.25) but also the greatest variability in usefulness ratings (average SD 1.61), reflecting more disagreement among participants.

These patterns suggest that questions focused on daily management or prognosis were perceived as clearer and more useful, while highly technical interpretation–related or medication-related questions generated more varied responses. Questions that combined interpretation with recommended action tended to show the widest dispersion, indicating that multi-intent prompts may be more difficult for patients to evaluate consistently.

### Summary of Patient Evaluation

In summary, patients rated the tailored LLM-generated questions as broadly understandable, with usefulness judged somewhat lower on average. Scenario-level results showed that the chronic kidney disease scenario questions were rated most favorably, while questions in the diabetes with liver disease scenario received the lowest ratings. Item-level analysis highlighted that most of the wide variation in ratings occurred within the hyperlipidemia with diabetes scenario, where some questions were rated highly by some participants but poorly by others. Category-level analysis suggested that lifestyle and prognosis questions were viewed as both clear and useful, whereas technical interpretation–related and medication-related questions generated lower or more variable evaluations. Together, these findings indicate that both the clinical scenario and the type of question shaped how patients perceived the clarity and value of LLM-generated prompts.

## Discussion

### Principal Findings

The primary aim of this study was to evaluate the feasibility of using LLMs to generate clinically relevant, patient-friendly follow-up questions based on laboratory test results contextualized within EHR data. Through a 3-round, user-centered iterative process involving both clinician and patient evaluations, we found that refined prompt engineering substantially improved the clarity, clinical relevance, and actionability of LLM-generated questions. Across rounds, nearly all questions were rated as clearly phrased by clinicians, and a high proportion were judged clinically valid. Notably, while GPT-4o and LLaMA 3.2 performed comparably on clarity, LLaMA 3.2–generated questions were rated more favorably on several measures of clinical validity and clinical appropriateness. In the final phase, patient evaluations confirmed that most questions were understandable but varied in perceived usefulness depending on scenario and question type. Lifestyle and prognosis questions were rated most favorably, while technical interpretation–related and medication-related questions showed more variability.

From a clinical standpoint, this work illustrates a feasible pathway for embedding AI-generated QPLs directly into patient portals to help patients formulate targeted questions ahead of their visits, particularly for individuals managing chronic conditions such as diabetes and chronic kidney disease, where routine laboratory monitoring often requires nuanced interpretation. Clinician evaluations highlighted that these tools may streamline previsit preparation and focus discussions on clinically urgent, actionable concerns, such as kidney function decline or elevated glucose, while reducing generic or tangential inquiries. When accurate and well-targeted AI-generated questions have the potential to enhance shared decision-making, improve treatment adherence, and reduce follow-up messaging burden caused by vague or incomplete patient inquiries. However, the variation in clinician ratings, reflected in low interrater reliability, suggests that perceptions of validity, appropriateness, and significance may be influenced by individual practice patterns. Therefore, AI-generated content may need to be reviewed or filtered through configurable templates that align with local clinical practice norms before patient-facing deployment, highlighting the need for standardized evaluation frameworks and clearer safety guardrails in future studies.

Patient ratings also differed across scenarios, with questions related to chronic kidney disease being evaluated most positively and those related to diabetes with liver disease least favorably. This suggests that both clinical complexity and question type shape patient perceptions and that refinements are needed to improve the usefulness of LLM outputs across diverse contexts. Although standard readability indices suggested moderate reading levels, higher Dale-Chall scores likely reflect the inclusion of necessary clinical terminology, which may not accurately capture patient comprehension in health care contexts. Notably, patient evaluation results indicated moderate to high understandability, suggesting that traditional readability metrics may overestimate difficulty for medically grounded content.

Although iterative prompt refinements substantially reduced hallucinations, residual inaccuracies were still observed in round 3. These primarily included the introduction of unsupported clinical assumptions, overgeneralized recommendations, and occasionally low-priority or loosely grounded questions. For example, some questions introduced conditions not explicitly present in the vignette (eg, “...especially considering my diabetes?”) or broadened interpretation beyond the provided data (eg, “Does this indicate poor nutrition, kidney disease, or another issue?”). In other instances, questions suggested additional testing without clear grounding (eg, “*...*should we check for vitamin D deficiency?”). Such cases were infrequent and limited to a small subset of generated questions, with no evidence of fabricated laboratory values or clearly unsafe clinical statements. Nevertheless, even low-frequency hallucinations may impact clinical reliability and user trust, underscoring the need for improved grounding, hallucination detection, and clinician-in-the-loop validation.

Our findings extend prior work on AI-assisted patient-clinician communication by shifting focus from generating responses to patient queries toward proactively generating targeted, context-aware questions that patients can bring to their clinicians. This approach aligns with evidence supporting QPLs as a means of improving patient engagement, recall, and satisfaction during clinical encounters. Similar to studies by Liu et al [16] and Gatto et al [17], we demonstrate that LLMs can meaningfully support the front end of patient-provider messaging workflows, potentially reducing ambiguity and improving consultation efficiency. However, unlike prior systems that primarily synthesized message content, our framework integrates structured clinical data such as abnormal laboratory results with LLM outputs to generate questions that are not only relevant but also personalized to the patient’s active diagnoses and medications. The observed differences between GPT-4o and LLaMA 3.2 suggest that model selection and prompt tuning remain critical determinants of clinical alignment. LLaMA’s higher ratings for relevance and significance, despite generating more complex language, indicate a possible trade-off between clinical depth and readability, an issue mirrored in prior work highlighting the importance of literacy-level calibration for patient-facing tools.

### Challenges of Using LLMs for Laboratory Question Generation

The earliest prompt iterations, while capable of producing relevant questions, occasionally generated hallucinations (eg, linking unrelated laboratory values and medications) and overgeneralized recommendations. Some outputs lacked sufficient specificity, reducing their utility in a focused clinical visit. These shortcomings were progressively mitigated through iterative refinement, explicit abnormal value references, and inclusion of condition- and medication-specific context. Despite improved outputs, limitations inherent to LLMs persist. Model-generated content can reflect training data biases, omit clinically critical nuances, or present outdated guidance. The observed differences between GPT-4o and LLaMA 3.2 further highlight variability in model behavior, necessitating ongoing evaluation and, in some cases, domain-specific fine-tuning. Additionally, low interrater reliability in clinician evaluations suggests that clinical relevance is partly subjective and may not be uniformly defined across specialties or settings.

Additional challenges include potential unintended consequences and health literacy misalignment. An important consideration in deploying AI-generated question prompts is the possibility of unintended effects on patient-clinician communication. If patients present questions that appear highly polished or technically sophisticated, clinicians may infer a higher level of health literacy than the patient actually possesses. Such inferences whether conscious or unconscious could result in explanations pitched at an overly technical level, reduced checking for understanding, or patient reluctance to ask follow-up questions due to embarrassment or perceived incongruence between the question’s sophistication and their own comprehension.

This potential mismatch echoes prior findings in health communication research showing that clinician assumptions about patient knowledge can influence explanation depth, language complexity, and engagement strategies. While the extent to which AI-generated question prompts amplify this risk remains an open empirical question, it underscores the importance of designing patient-facing AI systems that support and not mask patients’ informational needs. Potential mitigation strategies include pairing generated questions with explicit prompts encouraging clarification, incorporating health literacy–aware language constraints, and signaling to clinicians that questions may be AI assisted. Future studies should empirically examine how clinicians interpret AI-generated patient questions and whether such interpretations affect communication quality, comprehension, or patient comfort.

### Strengths and Limitations

A notable strength of this study is its mixed methods, iterative design involving both clinicians and patients in the evaluation loop. This approach allowed for systematic prompt refinement and ensured that usability considerations informed technical development. The use of real, deidentified EHR data to construct representative clinical profiles further strengthened ecological validity, while the comparison of 2 LLMs under matched conditions provided useful guidance for model selection in health IT deployments. However, several limitations merit discussion. First, our evaluation sample of clinicians was small, limiting the generalizability of interrater reliability findings. Second, although patient evaluations offered valuable insight into perceived clarity and usefulness, these were based on selected questions from hypothetical scenarios rather than actual care episodes, potentially limiting ecological realism. Although this approach was intended to balance clinical validity, content diversity, and participant burden, the resulting sample may not fully represent the complete distribution of LLM-generated outputs, including lower-quality or less appropriate questions. Consequently, the reported patient ratings of understandability and usefulness may be positively biased. Third, while prompt refinements reduced hallucinations, complete elimination was not achieved, and any residual inaccuracies could undermine patient trust if deployed at scale. Fourth, this study compared GPT-4o with a quantized LLaMA 3.2 3B model, which differ substantially in model capacity and reasoning power. This choice was intentional and driven by deployment considerations rather than an attempt to benchmark open-source versus closed-source models at an equivalent scale. Because the question generation module is intended for real-time, patient-facing use, it must support low-latency responses, efficient computation, and deployment in local or edge environments. Smaller open-source models such as LLaMA 3.2 3B better reflect these constraints, particularly for privacy-preserving workflows that avoid reliance on external APIs. As a result, our findings should not be interpreted as representative of the performance ceiling of open-source LLMs nor generalized to larger models (eg, LLaMA 3.1 70B) with substantially different computational and reasoning profiles. Future work will explore how question quality scales with model size under varying deployment constraints. Fifth, the patient evaluation was conducted only on selected questions generated by GPT-4o. Although clinician evaluations compared GPT-4o and LLaMA 3.2 in round 3, the patient-facing survey scenarios were based on questions generated for patients 2, 5, and 6, whereas LLaMA 3.2 was applied only to patients 7, 8, and 9. Therefore, the patient understandability and usefulness ratings should not be interpreted as comparative evidence between GPT-4o and LLaMA 3.2. Sixth, readability was assessed using standard, formula-based indices (eg, FKGL), which provide objective and reproducible estimates of linguistic complexity but may be suboptimal for evaluating health care text. Such metrics can penalize the use of precise medical terminology and may not fully capture whether increased complexity reflects reduced patient-friendliness or greater clinical accuracy. While we partially mitigated this limitation through direct patient ratings of understandability and usefulness, we did not apply health care–specific instruments such as the Patient Education Materials Assessment Tool (PEMAT) [22], which would require additional structured evaluation. Finally, although the *Introduction* emphasizes the challenges that older adults may face when interpreting laboratory results, the patient evaluator cohort included a broader adult population, with a mean age of 53.6 (SD 16.1) years, as shown in Table 10. As a result, the understandability and usefulness ratings may not fully reflect the perspectives of older adults, particularly those with lower health literacy, multiple chronic conditions, cognitive limitations, or other barriers to interpreting medical information. The patient evaluator sample was also unevenly distributed across the 3 scenarios. Scenario 3, which corresponded to patient #6 with both hyperlipidemia and diabetes, included only 3 participants; therefore, the means and SDs for this scenario should be interpreted as descriptive only and not as stable estimates of patient perceptions for this condition group. Finally, across the overall patient evaluator cohort, participants were highly educated, with many reporting college-, master’s-, or doctoral-level education. This educational skew in the full evaluation sample may have positively biased ratings of question understandability and limits the generalizability of the findings to patients with lower educational attainment or limited health literacy.

### Future Work

Future work should evaluate AI-generated QPLs using more comprehensive tools such as PEMAT to assess both understandability and actionability. Although our readability analysis showed that GPT-4o generated simpler outputs than LLaMA 3.2, future studies should further examine how to balance clinical specificity with patient comprehension, particularly for individuals with limited health literacy. Future studies should also directly compare personalized, AI-generated QPLs with generic static QPLs to determine whether context-aware question generation provides added value for patient comprehension, visit preparedness, communication efficiency, and clinical outcomes. Patient perceptions of outputs from different models should be evaluated using the same clinical scenarios and comparable question sets.

Several methodological limitations should also be addressed. This study used the most recent clinical data within a 6-month window and did not incorporate longer-term longitudinal trends, which may limit the contextual accuracy of generated questions. In addition, although prompts instructed models to rank questions by medical urgency, this prioritization was not formally validated against clinician-defined benchmarks. Future work should incorporate longitudinal patient data and systematically validate urgency ranking against expert consensus. Finally, future studies should include larger and more diverse patient samples, especially older adults, to better assess usability and perceived value in the target population. Integration of real-time LLM-generated QPLs into patient portals, combined with outcome evaluations, will be important for assessing effects on patient satisfaction, consultation efficiency, clinical decision quality, and long-term patient engagement.

### Conclusions

This study evaluated the feasibility of using LLMs to generate patient-centered QPLs grounded in EHR-derived laboratory data. Through 3 rounds of clinician-in-the-loop prompt refinement, the generated questions became more specific, actionable, and aligned with abnormal laboratory results, active diagnoses, and medications. Clinician ratings indicated high clarity for both GPT-4o and LLaMA 3.2, although ratings of clinical validity and usefulness (ie, clinical appropriateness, significance for the patient’s health, and willingness to answer) varied across clinicians and models. Patient evaluation of selected GPT-4o–generated questions suggested generally favorable understandability, with more variable ratings for perceived usefulness. These findings support the potential of clinician-reviewed LLM-generated question prompts as a communication aid for laboratory result interpretation while also highlighting the need for model-specific validation, readability optimization, broader patient testing, and clinician oversight before real-world implementation.

## Supplementary material

10.2196/87280 Multimedia Appendix 1 Example synthetic patient profile used for question generation.

10.2196/87280 Multimedia Appendix 2 First-, second-, and third-round prompts for the large language model (LLM) to generate questions; figures depicting clinician ratings for LLM-generated questions; and tables showing the number of questions in each rating range, ratings of individual tailored question items across clinical scenarios, and average patient ratings of LLM-generated questions grouped by primary category.

10.2196/87280 Multimedia Appendix 3 Evaluation of large language model–generated clinical laboratory test questions in round 3 reviewed by clinicians.

10.2196/87280 Multimedia Appendix 4 Item-level artificial intelligence–generated question evaluation results by patients.

## Abbreviations

CLAIR: Comprehensive Large Language Model Artificial Intelligence Responder
CUI: Concept Unique Identifier
EFLM: European Federation of Clinical Chemistry and Laboratory Medicine
eGFR: estimated glomerular filtration rate
EHR: electronic health record
HbA_1c_: hemoglobin A_1c_
HIPAA: Health Insurance Portability and Accountability Act
HITECH: Health Information Technology for Economic and Clinical Health
ICC: intraclass correlation coefficient
ICD-10: International Classification of Diseases, 10th revision
ICD-9: International Classification of Diseases, 9th revision
LLM: large language model
LOINC: Logical Observation Identifiers Names and Codes
PEMAT: Patient Education Materials Assessment Tool
QPL: question prompt list
