# Supplementary material for: Generating Question Prompt Lists From Electronic Health Record Data Using Large Language Models: Iterative Evaluation Study
Source: J Med Internet Res. 2026 Jul 9;28:e87280. doi: 10.2196/87280 (PMC13349228; doi:10.2196/87280)
Supplement: Multimedia Appendix 1 [file jmir-v28-e87280-s001.pdf]

Multimedia Appendix 1

Table S1. Example synthetic patient profile used for question generation. This table illustrates the structure of the synthetic profiles used in the study, including demographic information, laboratory results, medications, and diagnoses. The values are synthetic and are provided only to demonstrate the data format; they are not derived from or linked to any real patient.

Synthetic patient profile
I am a 63 year old white female.
The following are my latest lab test results:
Lab Test Name, Unit, Test Value
Sodium, mmol/L, 139.0
Potassium, mmol/L, 4.3
Chloride, mmol/L, 102.0
Carbon Dioxide, mmol/L, 24.0
Creatinine, mg/dL, 1.42
Blood Urea Nitrogen, mg/dL, 21.0
Glucose, mg/dL, 156.0
Hemoglobin A1c/Hemoglobin.total, %, 7.4
Cholesterol.total, mg/dL, 198.0
Cholesterol in HDL, mg/dL, 42.0
Cholesterol in LDL, mg/dL, 116.0
The following medications were recently used:
Drug_CUI, Medication
314076, lisinopril 10 MG Oral Tablet
860975, metformin hydrochloride 500 MG Oral Tablet
617314, atorvastatin 20 MG Oral Tablet
The following diagnoses were listed into my chart:
ICD Code, Diagnosis
E11.9, Type 2 diabetes mellitus, without complications
I10, Essential (primary) hypertension
E78.5, Hyperlipidemia, unspecified
N18.31, Chronic kidney disease, stage 3a

Note: All values shown in this appendix are synthetic. They were created to demonstrate the formatting and clinical structure of the patient profiles used for question generation. The profile should not be interpreted as representing an actual individual or as providing clinical advice.

Multimedia Appendix 1 presents an example of the synthetic patient profile format used for question generation. Each profile is structured as a short patient-facing summary and includes four main components: demographic information, recent laboratory test results, recently used medications, and diagnoses listed in the patient's chart. The example profile describes a synthetic 63-year-old female patient with laboratory values, medication records, and diagnosis codes related to diabetes, hypertension, hyperlipidemia, and chronic kidney disease.

The laboratory section includes the lab test name, measurement unit, and test value. The medication section includes the medication concept identifier and medication name, dose, and route when available. The diagnosis section includes ICD codes and corresponding diagnosis descriptions. These structured fields were designed to provide sufficient clinical context for generating patient-centered questions while maintaining a consistent format across profiles.
