# Supplementary material for: Generating Question Prompt Lists From Electronic Health Record Data Using Large Language Models: Iterative Evaluation Study
Source: J Med Internet Res. 2026 Jul 9;28:e87280. doi: 10.2196/87280 (PMC13349228; doi:10.2196/87280)
Supplement: Multimedia Appendix 2 [file jmir-v28-e87280-s002.pdf]

Multimedia Appendix 2

LLM Prompts for Question Generation

Textbox S1. Round 1 prompt for LLM to generate questions. The initial prompt instructed the large language model (LLM) to generate patient-centered questions based on de-identified clinical profiles containing demographics, laboratory test results, medications, and diagnoses.

What questions should I ask my physician based on the following clinical data to ensure my treatment plan is holistic, addresses all underlying conditions, and adapts to changes in my health status?

Please make sure to ask four questions for each lab test, considering the lab test values with reference ranges.

Only provide the questions to the physician.

Patient's Profile and Clinical Data:

{

[insert clinical profile]

}

Always adhere to the following instructions.

1. List the questions in order of priority, ranking them based on medical urgency.
2. Focus questions on actions the patient should take.
3. Give higher relevance to health-related questions.
4. Rank the questions according to the urgency of the existing conditions.
5. Generate questions that can be communicated to the physician within 15 minutes.
6. If the same lab test appears twice, generate four questions for both instances using the corresponding test values.

Textbox S2. Round 2 prompt for LLM to generate questions. The refined prompt instructed the large language model (LLM) to generate a list of 20 patient-centered clinical questions specifically targeting abnormal laboratory test results. The design emphasized prioritization by medical urgency, explicit identification of abnormal values, and alignment with the patient's medical history and current medications (e.g., diabetes, hypertension, insulin therapy).

Generate a list of 20 precise clinical questions for a patient to ask their clinician regarding abnormal lab test results. Follow these guidelines:

Guidelines:

Order of Questions: Rank questions by clinical urgency, starting with life-threatening concerns such as kidney function, glucose levels, and cardiovascular risks.

Highlight Abnormal Values: Explicitly mention which lab test values are abnormal and explain their significance in the question.

Medication and Disease Context: Align each question with the patient's medical history (e.g., diabetes, hypertension) and current medications (e.g., insulin detemir, diltiazem, levothyroxine).

Actionable Outcomes: Focus on specific steps the patient can take, including treatment adjustments, follow-ups, and lifestyle changes.

Patient-Friendly Language: Use 6th-grade reading level and patient-friendly terms.

Consistency: Maintain the sequence of lab tests as provided in the clinical data.

Time-Efficient: Limit to 20 questions to fit into a 15-minute discussion with the clinician.

Patient's Profile and Clinical Data:

{[insert clinical profile]}

Output:

Generate only the questions in plain text without extra context or numbering. Each question should:

Explicitly state abnormal lab values.

Mention the patient's diseases (e.g., Type 2 diabetes, kidney disease, peripheral vascular disease, hyperlipidemia).

Include relevant medications (e.g., insulin detemir, levothyroxine).

Focus on proactive actions the patient can take, such as adjusting medications or following up with specific tests.

The goal is to support informed discussions during the clinical visit by focusing on urgent and actionable health issues.

Focus on generating questions that address abnormal lab results, considering reference ranges, recent medications, and diagnostic information.

Questions should emphasize actionable management steps, treatment adjustments, proactive health measures, and specific follow-up recommendations.

The aim is to support patient-centered care and facilitate an informed discussion during the clinical visit.

Textbox S3. Round 3 prompt for LLM to generate questions. Round 3 prompt guided the LLM to generate 20 patient-friendly clinical questions specifically targeting abnormal laboratory test results. It required explicit mention of abnormal values, integration of patient conditions and medications, and prioritization of urgent concerns while maintaining a 6th-grade reading level.

Your task is to generate a list of 20 patient-friendly clinical questions for a patient to ask their clinician regarding abnormal lab test results.

Steps to Follow:
1. **Review the patient's clinical profile** to understand their general health background, including medical history and current medications.
2. **Analyze the lab test data** to identify abnormal values, clearly noting which values fall outside the reference ranges and understanding their clinical significance.
3. **Generate a list of 20 precise clinical questions** that address these abnormal lab results.
4. **Sort the questions by clinical urgency**, starting with potentially life-threatening concerns (e.g., kidney function, high glucose, cardiovascular risks) and progressing to less critical issues.
5. **For each question:**
   - Explicitly state the **abnormal lab test values** and why they are significant.
   - Reference the patient's **medical conditions** (e.g., Type 2 diabetes, kidney disease, peripheral vascular disease, hyperlipidemia) and **current medications** (e.g., insulin detemir, diltiazem, levothyroxine).
   - Focus on **actionable outcomes**, such as adjusting medications, scheduling follow-up tests, or implementing lifestyle changes.
6. **Maintain the sequence of lab tests** as provided in the clinical data and limit the output to exactly **20 questions**, to ensure comprehensive coverage for evaluation purposes. These questions are intended as a pre-visit question pool, from which a smaller, prioritized subset can be selected for discussion during a clinical visit.
7. **Use simple, 6th-grade reading level language** to ensure the questions are patient-friendly.
###Patient Clinical Profile
***
Output Format:
The output should be structured as follows:

`Lab test name \t Question`
Example Output:
```
Glucose My glucose levels were high on recent tests (229 mg/dL and 228 mg/dL). Given my Type 2 diabetes, should I adjust my insulin detemir dose or add other medications to control my blood sugar?
estimated glomerular filtration rate (eGFR) My estimated glomerular filtration rate (eGFR) is low (46 mL/min/1.73m² for Black and 40 mL/min/1.73m² for non-Black patients). Does this suggest kidney disease, and should we adjust any medications like diltiazem that might impact kidney function?
```
Examples of Highly Rated Questions:
- **Actionable:** *My triglyceride level is 163 mg/dL, which seems elevated. Should I make specific dietary changes or increase my exercise to lower this, considering my Type 2 diabetes and hyperlipidemia?*
- **Specific:** *My hemoglobin A1c is elevated at 7.6%, indicating poor blood sugar control. Can we discuss lifestyle or dietary changes to improve this, or consider different diabetes treatments?*
- **Medication-aware:** *The creatinine level is slightly high at 1.26 mg/dL. Given my kidney function decline, would you recommend any lifestyle or dietary changes, or additional testing for kidney health?*

Examples of Lower Rated Questions to Avoid:
- **Too vague:** *Is my chloride level of 105 mmol/L normal, or could it indicate dehydration or other electrolyte imbalances that need attention?*
- **Not actionable:** *My ALT and AST levels are both low (ALT at 11 IU/L and AST at 16 IU/L). Does this indicate any issues with my liver function, especially with my hyperlipidemia?*
- **Lacks context:** *My sodium and potassium levels are within range, but is there any specific diet or hydration advice to ensure these remain balanced, especially considering my hypertension?*

Final Notes:
- **Ensure all 20 questions strictly follow the requested format.**
- **Focus on lab test abnormalities and their implications.**
- **Avoid general symptoms-based questions (e.g., cough, cardiomyopathy) unless linked directly to lab abnormalities.**

Supplementary Figures

Figure S1 presents the average clinician ratings of LLM-generated questions across key evaluation criteria, aggregated from rounds 2 and 3. Panel (a) shows the binary ratings for two aspects: whether questions were "Clearly Phrased" and made "Clinical Validity." Each clinician evaluated 20 questions per round, where the maximum score is 20—equivalent to marking all questions as "yes." The scores for "Clearly Phrased" were uniformly high across all clinicians, indicating strong agreement on the linguistic clarity of the questions. In contrast, there was greater variability in the "Clinical Validity" scores, with Clinician 3 consistently rating questions lower than Clinicians 1 and 2, highlighting differences in how each clinician perceived the medical validity or appropriateness of the questions. Panel (b) summarizes average Likert-scale scores (range: 1–5) across three criteria: Willingness to Answer, Clinical Appropriateness, and whether it is significant for the patient's health. Clinicians 1 and 2 generally gave higher scores across all categories, while Clinician 3 rated the questions more critically, particularly on "Willingness to Answer" and "Significant for the Patient's Health." Error bars indicate standard error, showing minimal variability for Clinicians 1 and 2, but larger uncertainty in Clinician 3's responses. Together, these findings suggest strong consistency in perceived question clarity, but divergent assessments of clinical relevance, reinforcing the importance of incorporating multi-clinician feedback in LLM prompt optimization and evaluation.

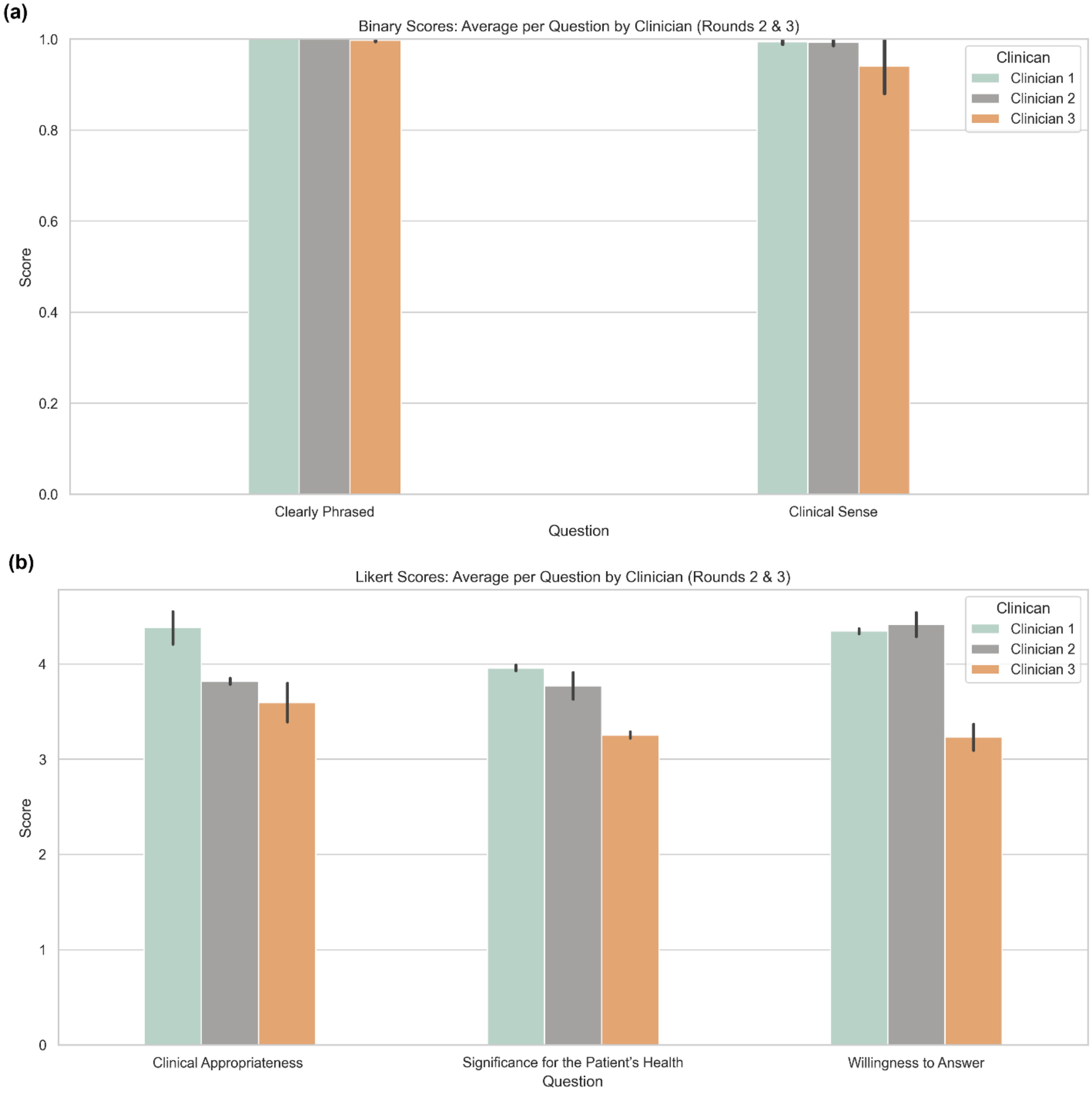

Figure S1. Average Clinician Ratings for LLM-Generated Questions Across Evaluation Criteria. (a) shows binary ratings for "Clearly Phrased" and "Clinical Sense" questions, aggregated across rounds 2 and 3. Panel (b) presents Likert-scale scores (1–5) "Clinical Appropriateness", "Significance for the Patient's Health", and "Willingness to Answer". Error bars represent standard error, and variations across clinicians highlight differing interpretations of question clarity and usefulness.

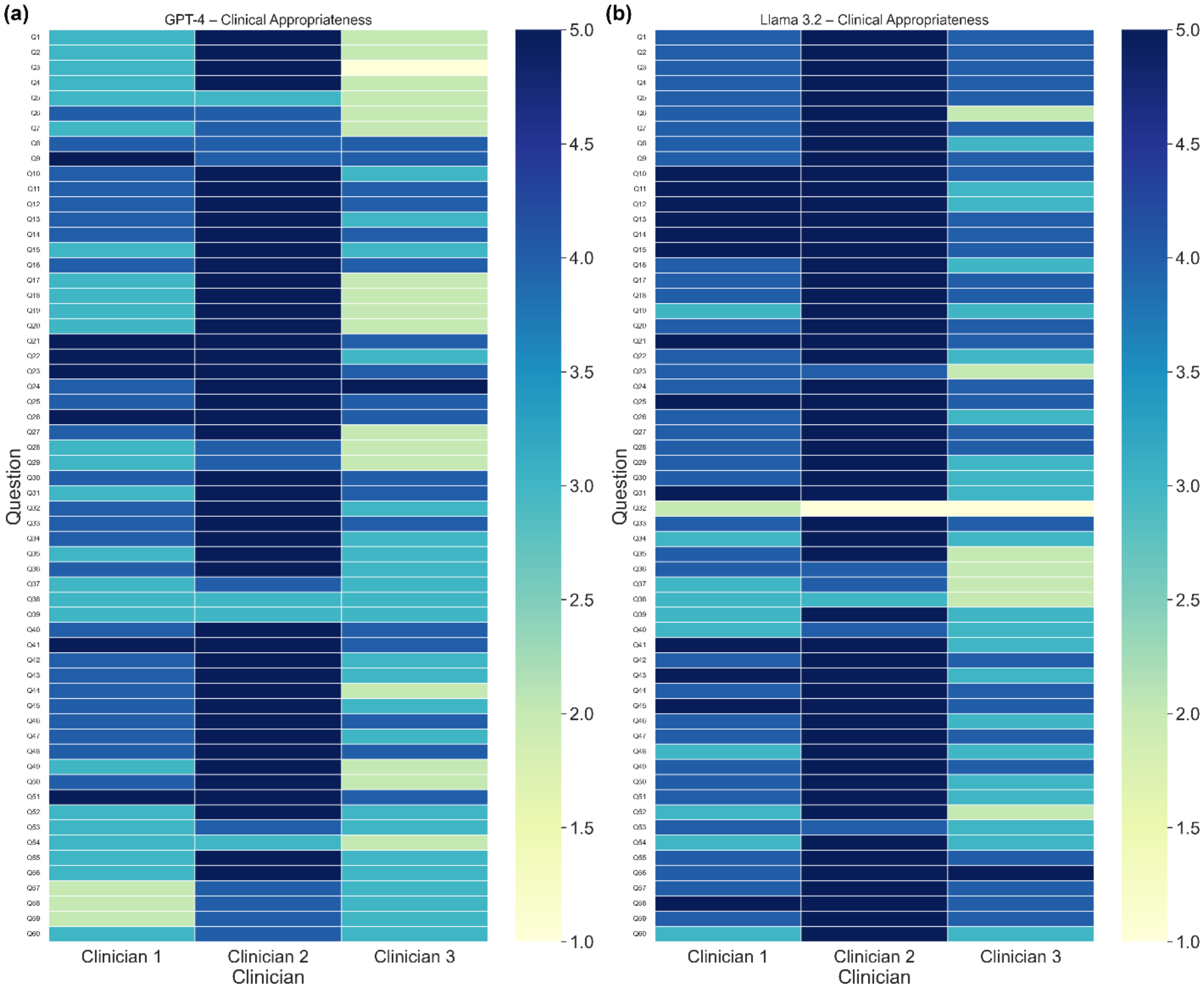

Figure S2. Clinician Ratings of LLM-Generated Clinical Questions in round 3 for the Likert Scale "Clinical Appropriateness": Comparison Between GPT-4o and LLaMA 3.2. (a) Ratings for GPT-4o-generated questions (Q1–Q60) were evaluated by three clinicians. (b) Ratings for LLaMA 3.2-generated questions (Q1–Q60) were evaluated by the same clinicians. Each cell represents a 1–5 Likert score (color-coded).

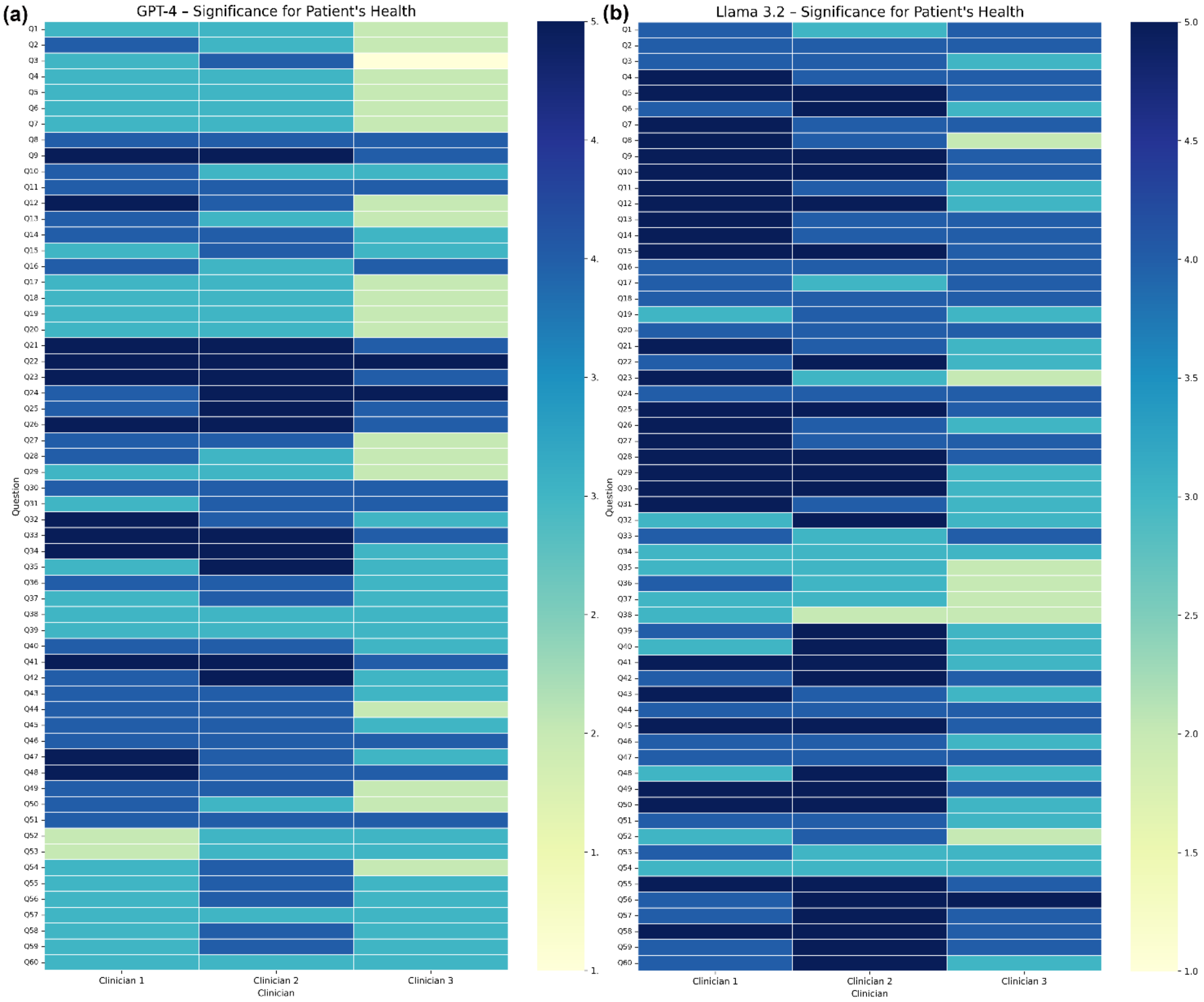

Figure S3. Clinician Ratings of LLM-Generated Clinical Questions in round 3 for the Likert Scale "Significance for Patient's Health": Comparison Between GPT-4o and LLaMA 3.2. (a) Ratings for GPT-4o-generated questions (Q1–Q60) were evaluated by three clinicians. (b) Ratings for LLaMA 3.2-generated questions (Q1–Q60) were evaluated by the same clinicians. Each cell represents a 1–5 Likert score (color-coded).

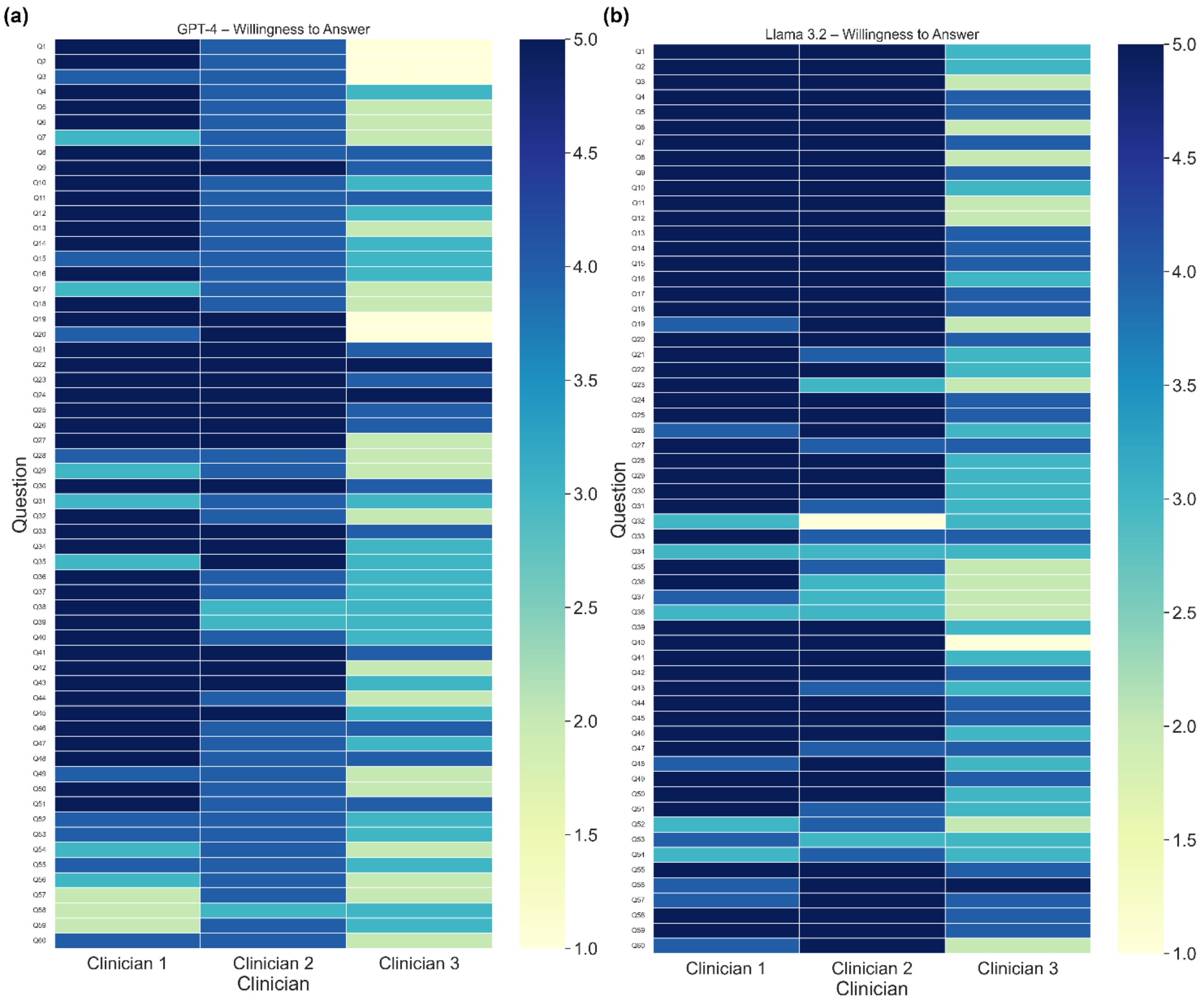

Figure S4. Clinician Ratings of LLM-Generated Clinical Questions in round 3 for the Likert Scale "Willingness to Answer": Comparison Between GPT-4o and LLaMA 3.2. (a) Ratings for GPT-4o-generated questions (Q1–Q60) were evaluated by three clinicians. (b) Ratings for LLaMA 3.2-generated questions (Q1–Q60) were evaluated by the same clinicians. Each cell represents a 1–5 Likert score (color-coded).

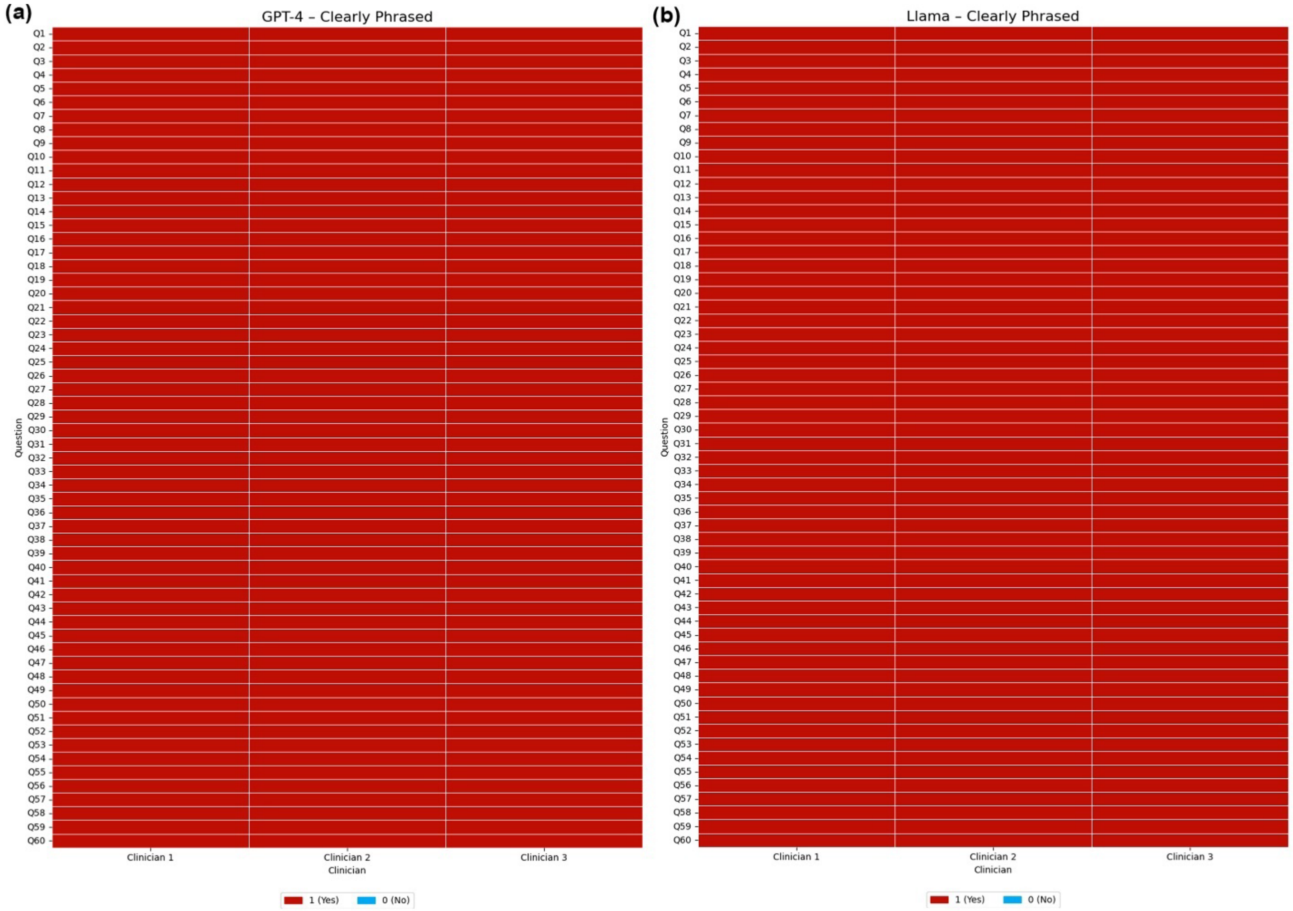

Figure S5. Clinician Ratings of LLM-Generated Clinical Questions in round 3 for the binary ratings for "Clearly Phrased": Comparison Between GPT-4o and LLaMA 3.2. (a) Ratings for GPT-4o-generated questions (Q1–Q60) were evaluated by three clinicians. (b) Ratings for LLaMA 3.2-generated questions (Q1–Q60) were evaluated by the same clinicians. Each cell represents a 0/1 binary score (color-coded).

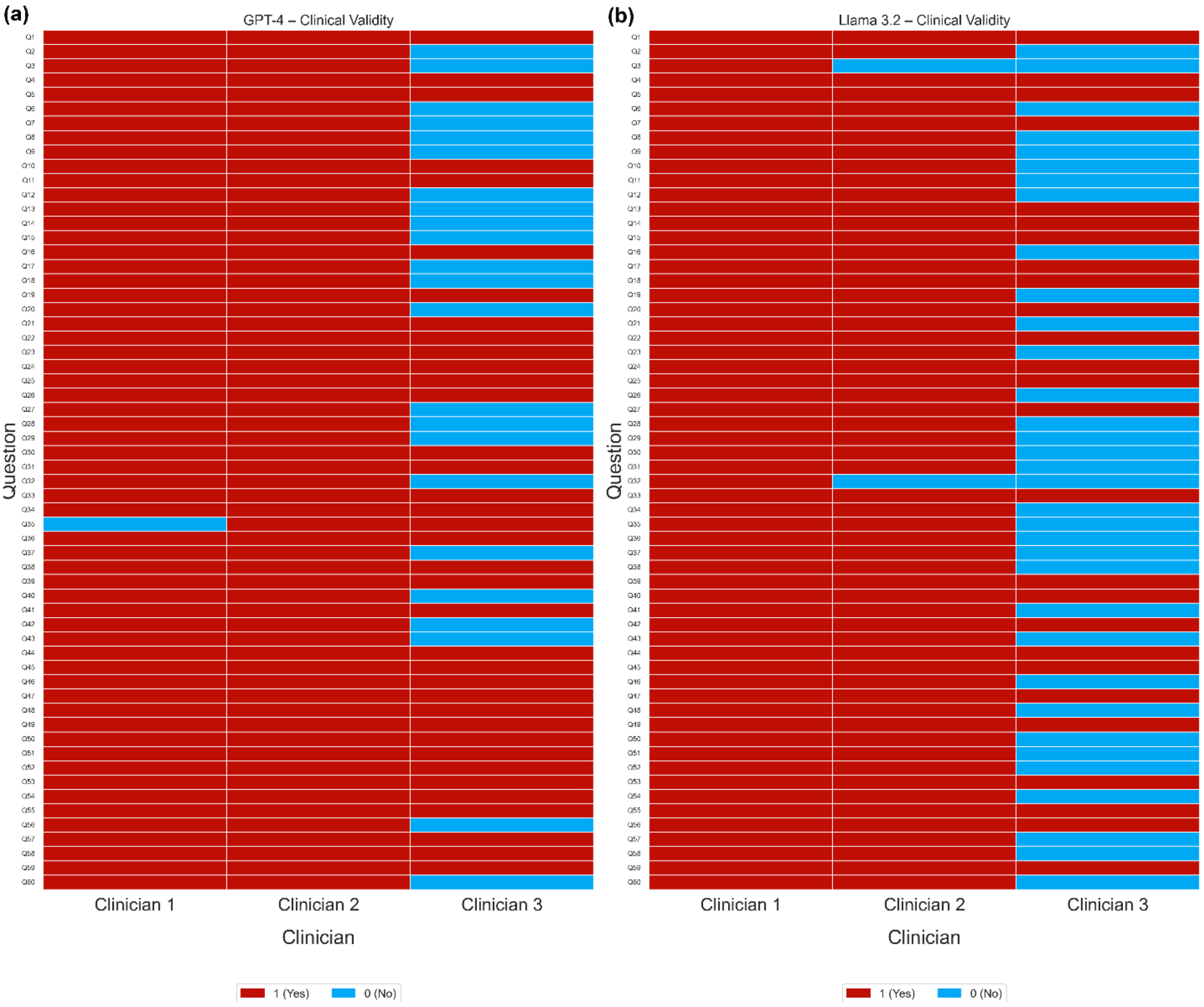

Figure S6. Clinician Ratings of LLM-Generated Clinical Questions in round 3 for the binary ratings for "Clinical Validity": Comparison Between GPT-4o and LLaMA 3.2. (a) Ratings for GPT-4o-generated questions (Q1–Q60) were evaluated by three clinicians. (b) Ratings for LLaMA 3.2-generated questions (Q1–Q60) were evaluated by the same clinicians. Each cell represents a 0/1 binary score (color-coded).

Supplementary Tables

Table S1. Number of questions in each rating range (understandability and usefulness)

AVERAGE RATINGS	Understandability (No. of Items)				Usefulness (No. of Items)			
	SCENARIO			TOTAL No. of Items	SCENARIO			TOTAL No. of Items
	1	2	3		1	2	3	
**High** (mean ≥4.0)	7	1	9	17	4	0	5	9
**Mid** (mean 3.5–3.9)	3	3	1	7	6	4	1	11
**Low** (mean <3.5)	0	6	0	6	0	6	4	10

Table S2. Ratings of individual tailored question items across clinical scenarios

	Understandability (Mean ± SD)			Usefulness (Mean ± SD)		
	SCENARIO 1	SCENARIO 2	SCENARIO 3	SCENARIO 1	SCENARIO 2	SCENARIO 3
**Q1**	4.32 ± 1.13	3.35 ± 1.28	4.67 ± 0.58	3.87 ± 1.19	3.37 ± 1.36	2.67 ± 2.08
**Q2**	4.41 ± 0.95	3.50 ± 1.29	4.67 ± 0.58	4.20 ± 0.94	3.56 ± 1.21	2.67 ± 2.08
**Q3**	4.46 ± 0.96	3.79 ± 1.29	4.67 ± 0.58	4.34 ± 0.83	3.73 ± 1.14	4.67 ± 0.58
**Q4**	4.10 ± 1.24	3.46 ± 1.31	4.67 ± 0.58	3.80 ± 1.17	3.83 ± 1.17	3.33 ± 1.53
**Q5**	3.72 ± 1.25	4.00 ± 1.05	4.33 ± 1.15	3.58 ± 1.36	3.92 ± 1.13	4.67 ± 0.58
**Q6**	3.86 ± 1.25	3.17 ± 1.38	3.67 ± 1.53	3.85 ± 1.14	3.40 ± 1.29	3.00 ± 1.73
**Q7**	4.05 ± 1.21	3.23 ± 1.37	5.00 ± 0.00	4.04 ± 1.02	3.06 ± 1.36	4.67 ± 0.58
**Q8**	3.89 ± 1.22	3.52 ± 1.24	4.67 ± 0.58	3.90 ± 1.14	3.31 ± 1.25	3.67 ± 1.15
**Q9**	4.04 ± 1.17	3.40 ± 1.40	4.33 ± 1.15	3.96 ± 1.24	3.27 ± 1.29	4.33 ± 0.58
**Q10**	4.37 ± 0.92	3.42 ± 1.36	4.33 ± 0.58	4.16 ± 1.08	3.31 ± 1.34	5.00 ± 0.00

Table S3. Average patient ratings of LLM-generated questions grouped by primary category

Primary Category	No. of Items	Understandability (Mean ± SD)	Usefulness (Mean ± SD)
Condition Impact	2	3.9 ± 0.15	4.01 ± 0.69
Lab Interpretation	9	3.68 ± 0.28	3.67 ± 0.65
Lifestyle / Self-Care	5	4.05 ± 0.33	3.77 ± 0.38
Medication Adjustment	4	3.96 ± 0.43	3.75 ± 0.25
Medication Safety	5	3.79 ± 0.49	3.52 ± 0.40
Prognosis / Next Steps	4	4.01 ± 0.55	3.76 ± 0.53
Tracking / Monitoring	1	4.1 ± NA	4.04 ± NA
